# For Weight Loss: Why Do GLP-1 Agonists Work, and How Can We Make Them Work Better?

**DOI:** 10.3390/biology15161395

**Published:** 2026-08-14

**Authors:** Charlise Giang, Jieyi Meng, Mahmoud Ali Mohammad, Zhenqi Liu, Xinle Wu, Yi Zhu

**Affiliations:** 1 USDA/ARS Children’s Nutrition Research Center, Department of Pediatrics, Baylor College of Medicine, Houston, TX 77030, USA; 2 College of Natural Sciences and Mathematics, University of Houston, Houston, TX 77004, USA; 3 Division of Endocrinology and Metabolism, Department of Medicine, University of Virginia Health System, Charlottesville, VA 22908, USA; 4 Research and Development Center, Sciwind Biosciences, San Ramon, CA 94583, USA; xinle.wu@sciwindbio.com

**Keywords:** GLP-1, GLP1RA, multi-receptor agonists, weight loss, obesity management

## Abstract

Glucagon-like peptide 1 receptor agonists (GLP-1RAs) have revolutionized the management of not only type 2 diabetes but also obesity. However, even with rapid therapeutic advances in GLP-1RAs, questions regarding the fundamental principles of GLP-1-mediated weight loss and the enhanced efficacy of next-generation therapies remain. In this comprehensive review article, we first examine the milestones of GLP-1, from its initial discovery and biological characterization to its successful translation into clinical therapy. Furthermore, this review highlights the latest clinical trial data, specifically HbA1c reduction, weight-loss efficacy, and safety profiles, while exploring current pipelines of major pharmaceutical companies. Additionally, we delineate the mechanistic principles underlying the efficacy of GLP-1 single- and multi-receptor agonists and address challenges in mitigating side effects of GLP-1RAs. Finally, we analyze the rapidly evolving landscape of next-generation, incretin-based combination therapies. With an abundance of GLP-1- related therapies already available or in development, we assess how future GLP-1 multi-receptor agonists can achieve enhanced efficacy and how leveraging weight-loss biology, such as set-point theory, can improve therapeutic outcomes.

## Simple Summary

Obesity is a critical public health issue that worsened during the late 20th century and persists today. While bariatric surgery remains an effective treatment, it is an invasive procedure, making pharmacological intervention a preferred option for many patients. Medications designed to mimic glucagon-like peptide-1 (GLP-1), a hormone that regulates appetite and blood sugar, were originally developed for diabetes treatment but have now revolutionized obesity treatment, producing weight loss almost comparable to that of surgery. However, GLP-1 receptor agonists (GLP-1RAs), alongside newer GLP-1 multi-receptor agonists, still have undesirable side effects, such as nausea and loss of lean muscle mass, as well as efficacy limitations that open doors for further improvement. This review seeks to identify lessons learned from GLP-1RA drug design and weight-loss biology to bridge the gap between scientific researchers and pharmaceutical companies in designing the next generation of GLP-1-related weight-loss medications. Advancing GLP-1RAs will offer millions of patients a safer, more effective path to weight management, alleviating the global burden of obesity and its comorbidities.

## 1. Introduction

Obesity has emerged as a global health pandemic since the latter half of the 20th century, with particularly high prevalence in the United States and other Western countries [1,2,3,4]. Obesity and its associated comorbidities, including hypertension, type 2 diabetes (T2D), and nonalcoholic fatty liver disease, have placed a massive burden on our healthcare system, underscoring the urgent need for effective pharmacological interventions [5,6,7]. Obesity is complicated by the profound heterogeneity of the disease and has been historically managed by behavioral modifications and caloric restriction. However, the long-term efficacy of these practices was largely unmet due to social and biological factors, underscoring the need for pharmacological interventions.

Glucagon-like peptide-1 (GLP-1) is a potent incretin hormone that is known to enhance meal-stimulated insulin release, inhibit the release of glucagon, slow gastric emptying, and decrease food intake [8,9,10]. Initially developed for T2D management, GLP-1 receptor agonists have shown clinically significant weight-loss benefits, establishing their role in obesity treatment. This remarkable efficacy, combined with approximately two decades of established safety [11], marks the first time a medication has approached the effectiveness of bariatric surgery for obese patients [12,13].

The body of literature for GLP-1 is extensive, with a great number of comprehensive reviews already available [9,10]. These reviews cover gene structure and expression [14,15], action on its receptor [16], animal studies of its mechanism of action [10], its development into anti-T2D therapy [8], further development into a blockbuster anti-obesity therapy [17], clinical trials demonstrating different GLP-1 agonists’ efficacy [11,18,19], side effects and meta studies of those clinical trials [11,18,19], cardiometabolic and renal outcomes of GLP-1 usage [20,21,22], and newer generations of GLP-1-based therapy [23].

This review aims to provide a comprehensive overview of glucagon-like peptide-1 (GLP-1), serving both as an accessible introduction for researchers new to the field and as a consolidated resource for experienced readers, while offering additional perspectives on strategies to improve GLP-1-based weight-loss therapies. We begin by summarizing fundamental aspects of GLP-1 biology, including its discovery, gene and protein products, mechanisms of action, and insights from animal studies. We then examine currently marketed GLP-1 receptor agonists, highlighting lessons learned from their clinical use with respect to efficacy and side effects, and present a comparative table summarizing their HbA1c-lowering and weight-loss efficacy at approved doses. Next, we review major ongoing efforts to develop next-generation GLP-1-based therapies, outlining the underlying rationale and strategies for their design. The review concludes with a discussion of weight-loss biology and how advances in this area can be leveraged to guide the development of more effective GLP-1-based drugs.

Distinct from previous reviews, we specifically address why weight-loss efficacy varies among GLP-1 receptor agonists (including multi-receptor agonists), how enhanced efficacy has been achieved, and how future research may further optimize therapeutic outcomes. Our goal is to engage both academic and industry researchers in exploring the mechanistic basis of GLP-1 agonist efficacy, the role of biomedical engineering in advancing these therapies, and the opportunities ahead for continuing to transform obesity drug development.

## 2. Methods

We have used PubMed, ClinicalTrials.gov, and official company pipeline updates to identify published clinical trials, published animal studies, conference abstracts, and press releases regarding GLP-1 receptor agonists from database inception through July 2026. For the trials selected in Table 1 and Table 2, inclusion criteria prioritized peer-reviewed trials that influenced the compound’s subsequent Food and Drug Administration (FDA) approval and highlighted the maximum therapeutic potential of these agents. Studies were also selected to represent participants with obesity without diabetes, type 2 diabetes without obesity, and/or obesity with other comorbidities. If no phase 3 peer-reviewed clinical trial data were available, phase 2 data were used. Retatrutide phase 3 results (TRIUMPH-1) are top-line and not yet peer-reviewed. Table 3 information on the company’s pipeline for obesity was sourced from the company’s official pages: Eli Lilly Pipeline (https://www.lilly.com/science/research-development/pipeline (accessed on 30 June 2026)) and Novo Nordisk Pipeline (https://www.novonordisk.com/science-and-technology/r-d-pipeline.html (accessed on 30 June 2026)). Data for Table 4 and Table 5 were sourced from published original research articles, clinical trials, and animal studies.

**Table 1:** HbA1c-lowering and weight-loss efficacy of FDA-approved GLP-1 receptor agonists and multi-receptor agonists, plus mazdutide (Approved in China) and retatrutide.

Drug & Highest Dose Evaluated	Trial Name	Population	Comparator	N (All Treatment Groups)	Duration	Baseline BMI/HbA1c (%)	Weight Loss (kg and %)	HbA1c Change (%)	Discontinuation Percentage (AE)	Citation/DOI
Exenatide (2 mg/wk)	DURATION-5	T2D	Exenatide twice daily	252	24 weeks	33/8.4	−2.3 kg (−2.4%)	−1.60%	5.00%	[24]
Liraglutide (1.8 mg/day)	LEAD-6	T2D	Exenatide	464	26 weeks	32.9/8.2	−3.24 kg (−3.5%)	−1.12%	Not Reported	[25]
Liraglutide (3 mg/day)	SCALE	Overweight/Obesity	Placebo	3731	56 weeks	38.3/5.6	−8.4 kg (−7.9%)	−0.30%	9.90%	[26]
Dulaglutide (1.5 mg/wk)	AWARD-1	T2D	Exenatide/Pioglitazone/Metformin	976	52 weeks (weight measured at 26 weeks)	33/8.1	−1.3 kg (−1.4%)	−1.51%	3.00%	[27]
Dulaglutide (1.5 mg/wk)	AWARD-3	T2D	Metformin	807	52 weeks (weight measured at 26 weeks)	34/7.6	−2.29 kg (−2.5%)	−0.70%	5.20%	[28]
Lixisenatide (20 μg/day)	GetGoal-X	T2D	Exenatide	634	24 weeks	33.7/8.03	−2.96 kg (−3.1%)	−0.79%	10.40%	[29]
Semaglutide (2.4 mg/wk)	STEP-1	Overweight/Obesity without T2D	Placebo	1961	68 weeks	37.8/5.7	−15.7 kg (−14.9%)	−0.45%	7.00%	[12]
Semaglutide (50 mg/day oral)	PIONEER PLUS	T2D	Semaglutide 14 mg	1006	52 weeks	33.7/8.9	−8.0 kg (−8.5%)	−2.00%	13.00%	[30]
Semaglutide (50 mg/day oral)	OASIS-1	Overweight/Obesity without T2D	Placebo	667	68 weeks	37.3/5.6	−15.5 kg (−15.1%)	−0.20%	6.00%	[31]
Tirzepatide (15 mg/wk)	SURPASS-1	T2D	Placebo	478	40 weeks	31.5/7.85	−9.5 kg (−11.1%)	−2.07%	7.00%	[32]
Tirzepatide (15 mg/wk)	SURMOUNT-1	Overweight/Obesity without T2D	Placebo	2539	72 weeks	38.2/5.6	−22.1 kg (−20.9%)	−0.51%	6.20%	[13]
Tirzepatide (15 mg/wk)	SURMOUNT-2	Overweight/Obesity and T2D	Placebo	938	72 weeks	35.7/8.07	−14.6 kg (−14.7%)	−2.08%	7.00%	[33]
Tirzepatide (15 mg/wk)	SURMOUNT-5	Obesity/Overweight with Comorbidities	Semaglutide 2.4 mg	705	72 weeks	39.4/5.6	−22.8 kg (−20.2%)	−0.50%	6.10%	[34]
Mazdutide (6 mg/wk)	GLORY-1	Overweight/Obesity without T2D	Placebo	610	48 weeks	31.3/5.5	−12.2 kg (−14.01%)	−0.31%	0.50%	[35]
Retatrutide (12 mg/wk)	Phase 2 Obesity	Overweight/Obesity without T2D	Placebo	338	48 weeks	37.4/5.5	−26.1 kg (−24.2%)	Not Reported	16%	[36]
Retatrutide (12 mg/wk)	Phase 2 T2D	Overweight/Obesity and T2D	Placebo/Dulaglutide	281	36 weeks	35.5/8.3	−17.18 kg (−16.94%)	−2.16%	15.00%	[37]
Retatrutide (12 mg/wk)	TRIUMPH-1 (Top-line results, not yet peer reviewed)	Overweight/Obesity with Comorbidities	Placebo	2339	80 weeks	Not Reported	−31.9 kg (−28.3%)	Not Reported	11.30%	https://investor.lilly.com/news-releases/news-release-details/lillys-triple-agonist-retatrutide-delivered-powerful-weight-loss (accessed on 30 June 2026)

## 3. Discovery of GLP-1

The search for incretin hormones began with the discovery of gastric inhibitory peptide (GIP). In 1969–1970, John Brown, who spent a sabbatical year in the laboratory of Victor Mutt in Stockholm, purified GIP from porcine intestine using classical peptide biochemistry [38,39,40]. Initially described as an inhibitor of gastric acid secretion, GIP was soon recognized to stimulate glucose-dependent insulin secretion, establishing it as the first incretin hormone [38,39,41]. However, neutralization of GIP did not abolish the incretin effect, suggesting that an additional insulinotropic factor remained unidentified [42].

The search for this second incretin turned toward intestinal endocrine L cells, known to produce glicentin, a 69-amino acid peptide containing the entire glucagon sequence at positions 33–61 [43,44]. Glicentin was identified as a product of the proglucagon precursor, but the proglucagon molecule was longer than glicentin, leaving open questions about the remaining sequences, collectively termed the major proglucagon fragment (MPGF) [45,46,47]. These observations prompted investigation into the broader processing of proglucagon and the biological significance of its additional peptides.

In the early 1980s, sequencing of anglerfish proglucagon revealed a glicentin-like peptide at the N-terminus and an additional glucagon-like sequence at the C-terminus [48]. Shortly thereafter, mammalian proglucagon was shown to contain two related peptides, glucagon-like peptide-1 (GLP-1) and glucagon-like peptide-2 (GLP-2) [45,49,50]. Functional studies demonstrated that GLP-1 was a potent insulin secretagogue with stronger incretin activity than GIP, while GLP-2 mainly had intestinal growth effects [51,52]. In addition, GLP-1 suppressed glucagon secretion, directly addressing the dual defects of impaired insulin secretion and excess glucagon action characteristic of T2D [51]. These discoveries laid the foundation for the development of GLP-1 for T2D.

## 4. Biology of GLP-1

**Figure 1:**
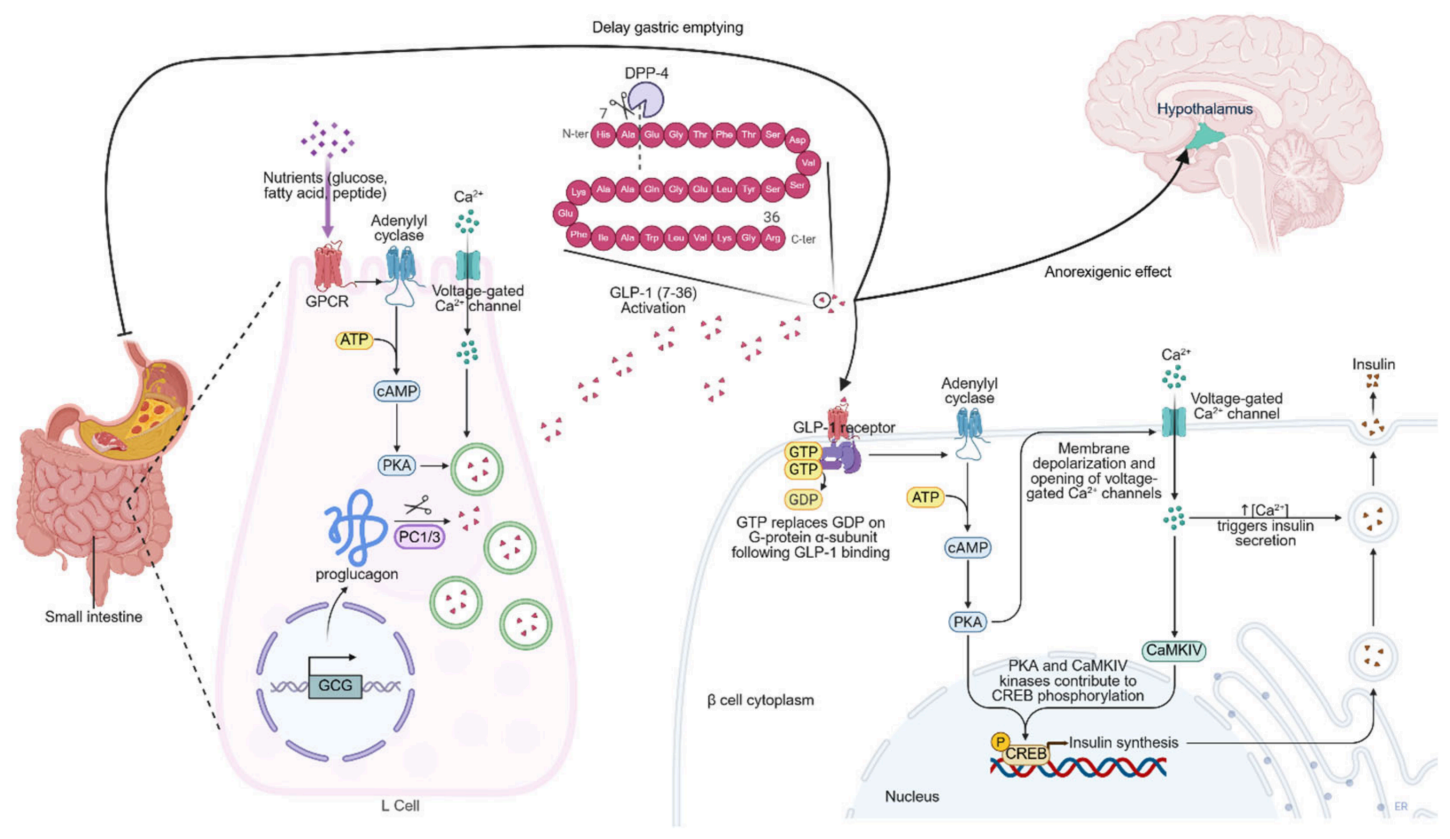
GLP-1 synthesis, signaling, and systemic metabolic regulation. GLP-1 is synthesized from the proglucagon (GCG) gene in intestinal L cells and secreted in a biphasic pattern following nutrient ingestion. Native GLP-1 (7–36) is rapidly inactivated by dipeptidyl peptidase-IV (DPP-4)-mediated cleavage at the N-terminus, resulting in a short half-life of 1–2 min. GLP-1 binding to the GLP-1 receptor activates adenylate cyclase, increasing cAMP and PKA/Epac2 pathways. Membrane depolarization triggers voltage-gated calcium channels, increasing intracellular calcium concentrations and triggering insulin secretion. PKA and calcium/calmodulin-dependent protein kinase type IV (CaMKIV) help contribute to CREB phosphorylation and insulin synthesis. GLP-1 also mediates anorexigenic effects via the hypothalamus and reward regions to reduce food intake, as well as the gastrointestinal tract to inhibit gastric emptying.

The potent insulinotropic properties and therapeutic potential of GLP-1 have stimulated extensive research into its biology (Figure 1). GLP-1 is derived from the proglucagon gene, whose protein product undergoes tissue-specific post-translational processing in intestinal enteroendocrine L cells, whereas in pancreatic α cells, proglucagon is primarily cleaved into glucagon. Most biologically active GLP-1 exists as GLP-1 (7–36) amide or GLP-1 (7–37) [47,53]. These peptides are secreted into the circulation in response to nutrient ingestion, with fat and carbohydrates being the principal physiological stimuli. GLP-1 release follows a biphasic pattern: an early phase, which is primarily mediated by vagal afferents that are activated by distension of the stomach and proximal intestine after ingestion of a meal [54,55], and a later, more sustained phase driven by direct nutrient sensing by L cells [52].

GLP-1 (7–36) amide is generally considered the predominant active form at around 80%, yet it has a remarkably short plasma half-life of ~1–2 min due to rapid degradation by the ubiquitous enzyme dipeptidyl peptidase-4 (DPP-4) and subsequently by neutral endopeptidase (NEP) [56]. Cleavage at the N-terminal yields GLP-1 (9–36) amide and GLP-1 (9–37), metabolites generally regarded as inactive [53]. DPP-4 is highly expressed on the luminal surface of endothelial cells lining capillaries and venules that drain the intestinal mucosa; because these vessels lie in close proximity to sites of GLP-1 secretion, the majority of secreted GLP-1 is inactivated before reaching the systemic circulation [57]. As a result, only a small fraction of intact GLP-1 escapes hepatic extraction and becomes available to act on distant target tissues.

GLP-1 exerts its biological effects through the GLP-1 receptor (GLP-1R), a class B G protein-coupled receptor (GPCR) characterized by an extracellular N-terminal domain critical for ligand recognition, seven transmembrane α-helices, and an intracellular C-terminal tail [58,59]. GLP-1R is broadly expressed across multiple tissues, including pancreatic islets, the gastrointestinal tract, lung, heart, kidney, and the nervous system [60]. Upon ligand binding, GLP-1R undergoes a conformational change that activates heterotrimeric G proteins, stimulating adenylate cyclase and driving intracellular cAMP accumulation. Elevated cAMP activates protein kinase A (PKA), which enhances insulin synthesis and secretion in pancreatic β cells while suppressing glucagon release from α cells [61,62,63]. In parallel, cAMP activates the guanine nucleotide exchange factor Epac2, which regulates insulin granule exocytosis via the small GTPase Rap1 [64]. Epac2 also lowers the ATP threshold required for closure of K_ATP_ channels, thereby promoting β-cell depolarization, opening of voltage-dependent Ca^2+^ channels, Ca^2+^ influx, and amplification of insulin secretion [65,66].

Beyond G protein-dependent signaling, GLP-1R activation also recruits β-arrestins, multifunctional scaffold proteins that extend signaling beyond the canonical cAMP–PKA axis [62]. β-arrestin-mediated pathways activate kinases such as ERK1/2 and transcription factors including CREB, which regulate gene expression programs linked to β-cell proliferation and function [62,67]. Acutely, β-arrestin recruitment augments GLP-1–stimulated insulin secretion, but under conditions of prolonged receptor activation, it contributes to receptor desensitization and downregulation, ultimately limiting insulinotropic responses [68].

Because GLP-1 is coded by the pre-proglucagon gene, which also encodes GLP-2 and glucagon, no “GLP-1-only” knockout (KO) in the strict genetic sense has been made. As a result, dissecting the role of GLP-1 in different metabolic organs heavily relies on studies employing tissue-specific GLP-1R KO mice. Whole-body GLP-1R KO mice display impaired oral glucose tolerance and lack the first phase of insulin secretion in vivo compared to wild-type mice, highlighting the importance of intestinally released GLP-1 in glucose-stimulated insulin secretion (GSIS) [69]. GLP-1’s direct action on pancreatic β cells to stimulate insulin release is further confirmed using β cell-specific GLP-1R KO mice [70]. This provided the rationale for developing GLP-1 analogs to treat type 2 diabetes, especially at a time when non-glucose-stimulated insulin secretagogues such as sulfonylureas were widely used.

Beyond its role in glucose control, GLP-1 potently reduces food intake by acting through both peripheral and central pathways. A major early mechanism is the inhibition of gastric emptying, which delays nutrient delivery to the small intestine, prolongs gastric distension, and enhances satiation. This effect is primarily mediated through peripheral GLP-1 receptors on vagal afferent neurons and enteric neurons, rather than by direct GLP-1 action inside the brain. In rodents, peripheral GLP-1 activates vagal afferents that project to the nucleus tractus solitarius (NTS), where second-order neurons integrate gastrointestinal signals to suppress meal size [71]. In parallel, central GLP-1 receptors located in the NTS, hypothalamic nuclei (arcuate nucleus, paraventricular nucleus pathway), and mesolimbic reward regions (ventral tegmental area, nucleus accumbens) modulate feeding behavior through homeostatic and hedonic pathways [72,73]. Genetic or pharmacologic blockade of GLP-1 receptors in these brain regions increases food intake and disrupts normal meal patterns [74,75]. Moreover, GLP-1 actions in mesolimbic areas selectively reduce consumption of palatable foods without affecting bland chow intake. Together, these mechanisms, peripheral vagal–brainstem signaling controlling gastric emptying and central GLP-1 actions controlling satiety and reward, explain how GLP-1 reduces feeding and supports weight loss. In humans, GLP-1 receptor agonists have been shown by fMRI to reduce anticipatory food reward and activity in reward-related brain regions, suggesting a mechanism for preventing overeating and supporting sustained weight loss [76,77]. Taken together, these central and peripheral mechanisms explain how GLP-1R agonists exert appetite-suppressing effects that translate into clinically meaningful weight reduction.

## 5. Development of GLP-1 into Therapy

**Figure 2:**
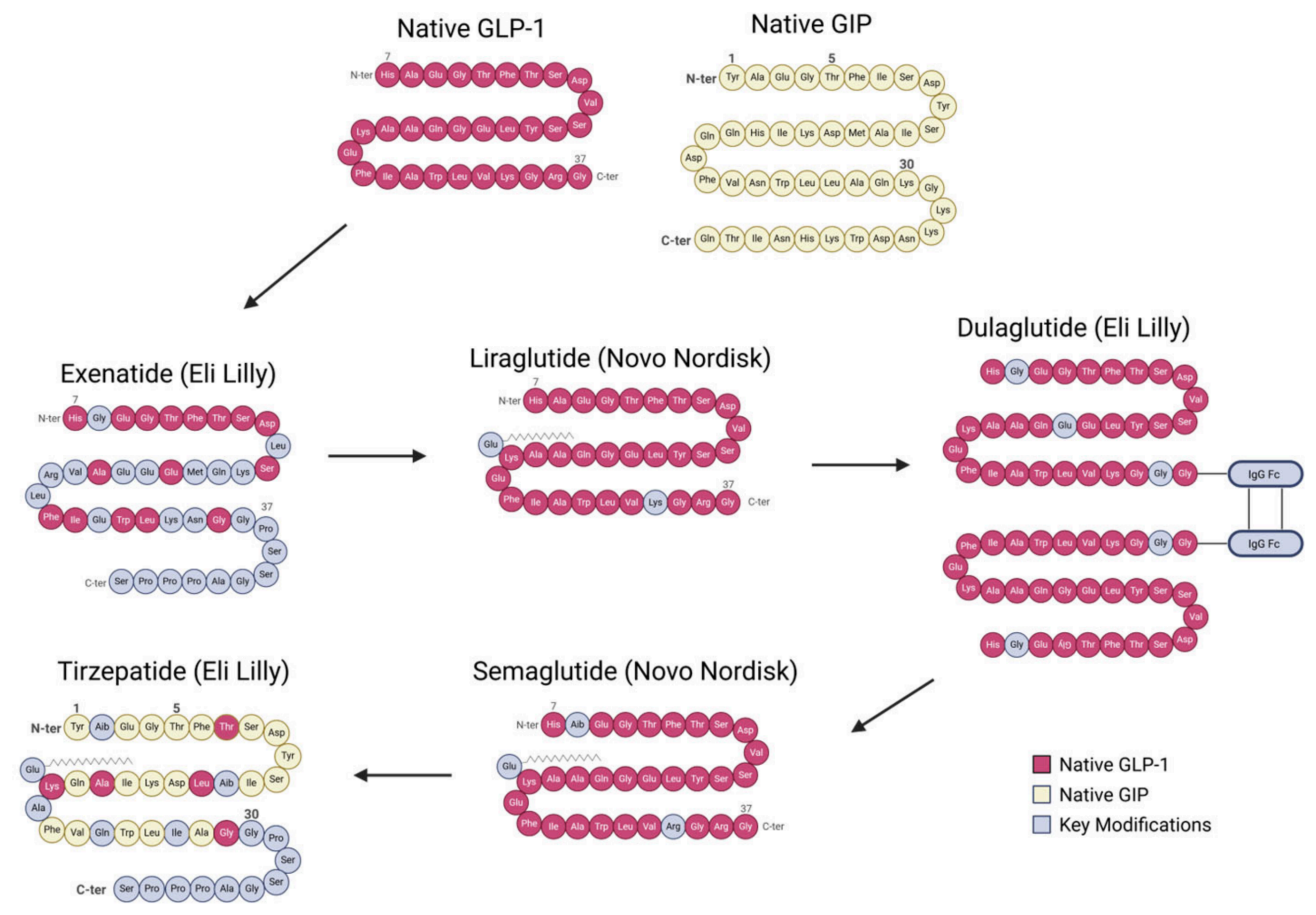
Structural evolution of FDA-approved GLP-1 receptor (GLP-1R) agonists. In 2005, Byetta (exenatide) was the first GLP-1R agonist approved as an anti-type 2 diabetes (T2D) drug [85]. In 2014, Saxenda (liraglutide) was the first GLP-1R agonist approved specifically for weight loss in the United States [86]; at a dose of 3.0 mg/day, it leads to an average loss of about 8% of overall body mass. The modification with a C16 fatty acid chain allows for reversible albumin binding, extending the half-life to 13 h for once-daily dosing. Trulicity (dulaglutide), made by Eli Lilly, was approved for T2D in 2014 for once-weekly dosing. The integration of two GLP-1 analogs linked by an Fc fragment of IgG4 significantly increased molecular size to reduce renal clearance [27]. In 2021, Wegovy (semaglutide), made by Novo Nordisk, received FDA approval for the same indication based on data showing individuals taking semaglutide (up to 2.4 mg per week) lose an average of 15% of their body weight [12]. Further modification with a C18 fatty acid chain and an Aib substitution increased albumin affinity and DPP-4 resistance, allowing for once-weekly administration. Tirzepatide (Eli Lilly), a first in class GLP-1R/GIPR dual agonist, demonstrated a mean weight loss of up to 20.9% for the 15 mg dosage treatment [13]. Specific amino acid substitutions allow for dual GLP-1R and GIPR activity, while incorporation of a C20 fatty acid extends its half-life through albumin binding, optimizing impact on glucose and weight regulation.

The short biological half-life and rapid renal clearance of native GLP-1 posed major challenges for its therapeutic development. In addition, the high incidence of nausea and vomiting observed in early trials with GLP-1 receptor agonists nearly derailed their advancement. To overcome these obstacles, researchers focused on designing GLP-1 analogs with increased resistance to DPP-4 degradation and improved pharmacokinetic stability (Figure 2). At the same time, clinical trial strategies such as gradual dose titration were introduced, which have substantially reduced gastrointestinal side effects and enabled the successful development of GLP-1-based therapies.

The first GLP-1 receptor agonist was exenatide, which was approved by the Food and Drug Administration in April 2005 for the treatment of type 2 diabetes. In the early 1990s, researchers studying the Gila monster (*Heloderma suspectum*) identified a peptide in its venom called exendin-4 [78]. This peptide shares ~53% sequence identity with human GLP-1, fully activates the human GLP-1 receptor, but is resistant to degradation by DPP-4, giving it a much longer plasma half-life (~2–4 h vs. GLP-1’s ~2 min) [78,79]. Amylin Pharmaceuticals licensed and developed synthetic exendin-4 as exenatide. In 2002, Amylin partnered with Eli Lilly to co-develop and commercialize the drug, which became the first GLP-1 receptor agonist approved for type 2 diabetes in 2005 (marketed as Byetta^®^, a twice-daily injection). Its success paved the way for the development of longer-acting GLP-1 analogs.

Liraglutide was developed at Novo Nordisk in the 1990s. The team focused on chemical modifications to enhance stability and prolong circulation time by attaching a C16 palmitic acid chain via a glutamic acid spacer to Lys26 of GLP-1 [80,81]. This modification promotes reversible binding to plasma albumin, extending liraglutide’s half-life to approximately 13 h, while also protecting it from DPP-4 degradation and reducing renal clearance [80]. The resulting analog, liraglutide, suitable for once-daily injection, retained full GLP-1 receptor activity and was approved by the FDA in 2010 for the treatment of type 2 diabetes under the brand name Victoza^®^ [82].

To improve upon the daily injections of liraglutide, Eli Lilly launched dulaglutide, with GLP-1 (7–37) covalently linked to an Fc fragment of human IgG4, which is administered once a week [27]. Dulaglutide was marketed under the brand name Trulicity and was approved by the U.S. FDA in 2014 as a once-weekly GLP-1 receptor agonist for adults with type 2 diabetes.

Semaglutide was developed at Novo Nordisk by substituting alanine with 2-aminoisobutyric acid at position 8 (to confer resistance to DPP-4 degradation) and attaching a C18 fatty diacid via a spacer to Lys26 (to enhance albumin binding). These modifications give semaglutide a plasma half-life of approximately 7 days, enabling once-weekly subcutaneous administration [83]. It was approved by the FDA in 2017 under the brand name Ozempic^®^ for type 2 diabetes, with clinical trials demonstrating superior HbA1c and body-weight reductions compared to earlier GLP-1 agonists. A novel oral formulation using an absorption enhancer (sodium N-[8-(2-hydroxybenzoyl)amino] caprylate, SNAC) was later developed, resulting in the first oral GLP-1 receptor agonist, approved in 2019 as Rybelsus^®^. In 2021, semaglutide was further approved as Wegovy^®^ for chronic weight management, based on its robust effects on weight reduction and cardiometabolic risk factors.

Despite the clinical success of GLP-1 receptor agonists in improving glycemic control and promoting weight loss, their effectiveness varies among patients. Bariatric surgery remains the most effective and durable treatment for severe obesity [84], but its high cost and invasive nature limit widespread adoption. In addition, gastrointestinal side effects, most notably nausea and vomiting, can reduce tolerability and require gradual dose titration during treatment initiation. These limitations have led to high rates of treatment discontinuation and prompted the search for next-generation incretin-based therapies. Building on the foundation of GLP-1 agonist development, researchers have advanced dual and triple agonists that combine GLP-1 activity with that of other hormones to engage multiple receptor pathways in metabolic tissues. This strategy aims to achieve superior efficacy in weight reduction and metabolic control compared to GLP-1 monotherapy.

## 6. GLP-1 Dual Agonists and Triagonists

Unimolecular multi-receptor agonists (MRAs) have emerged as highly effective treatments for glycemic control and weight loss. Combining the actions of GLP-1 with other peptides such as GIP or glucagon amplifies weight reduction beyond what is achieved with GLP-1 alone. Two dual agonists have been approved in the U.S. and China.

Tirzepatide is an FDA-approved once-weekly injectable peptide engineered from a GIP backbone and designed to activate both the GLP-1 and GIP receptors [87]. Its affinity for the GIP receptor is comparable to native GIP, while its affinity for the GLP-1 receptor is approximately five-fold lower than native GLP-1 [87]. It was developed by Eli Lilly and approved by the FDA for T2D and weight loss under the brand names Mounjaro^®^ and Zepbound^®^ in 2022 and 2023, respectively. GIP, an incretin hormone secreted by intestinal K cells, mediates much of the insulinotropic incretin effect in humans and exhibits both glucagonotropic and insulinotropic properties in a glucose-dependent manner. Tirzepatide combines the actions of GLP-1 and GIP to enhance the effects and/or tolerance of GLP-1 and broaden its therapeutic benefits [87]. In the SURMOUNT-1 phase 3 trials, patients achieved a mean weight loss of −15%, −19.5% and −20.9% in the 5 mg, 10 mg, and 15 mg groups after 72 weeks, respectively [13]. Additionally, the recent SURMOUNT-5 trial provided direct head-to-head evidence in adults who were overweight or obese without diabetes, demonstrating that tirzepatide treatment (at its maximum tolerated dose of 10–15 mg) yielded a significantly greater mean weight reduction (20.2%) than semaglutide (1.7–2.4 mg), which yielded a weight loss of 13.7% [34].

GLP-1/glucagon dual agonists aim to harness the anorectic and thermogenic effects of GLP-1 and glucagon, respectively, with glucagon receptor activation providing additional benefits on lipid metabolism and fatty acid oxidation [88]. Mazdutide, developed by Eli Lilly and Innovent Biologics, is an oxyntomodulin analog and GLP-1/glucagon dual agonist that has demonstrated clinically meaningful weight reduction [35]. In a 48-week trial, participants achieved body weight decreases of 11.0% in the 4 mg group and 14.0% in the 6 mg group [35]. Adverse effects were primarily gastrointestinal and occurred during dose escalation [35]. Based on these results, mazdutide was approved for T2D and weight loss in China in 2025. However, current trials are limited by the exclusion of patients with type 2 diabetes and a lack of participant diversity, limiting generalizability of these findings [35]. To address these gaps, Eli Lilly has initiated clinical trials in the United States to evaluate mazdutide’s efficacy in more diverse populations (NCT06124807).

Retatrutide is a 39-amino acid single peptide engineered from a GIP backbone to achieve triple agonist activity at the glucagon, GIP, and GLP-1 receptors [89]. It is 2.9 times less potent than human glucagon and 2.5 times less potent than GLP-1, but 8.9 times more potent than human GIP [89]. To provide resistance to DPP-4 cleavage, an Aib2 (α-amino isobutyric acid) substitution is incorporated into the peptide [89]. In preclinical studies, retatrutide induced greater body-weight loss than tirzepatide in obese mice when administered daily at 10 nmol/kg [90]. In a 48-week phase 2 obesity trial, participants achieved remarkable weight reductions of 22.8% and 24.2% with the 8 mg and 12 mg doses, respectively [36].

GLP-1 receptor agonists (e.g., semaglutide) and multi-receptor agonists (e.g., tirzepatide) are approved for chronic weight management as an adjunct to lifestyle intervention in adults with obesity (BMI ≥ 30 kg/m^2^) or overweight (BMI 27–29.9 kg/m^2^) who have at least one obesity-related comorbidity, such as type 2 diabetes, hypertension, dyslipidemia, obstructive sleep apnea, or established cardiovascular disease [12,13]. In contrast, these agents are approved for glycemic management in adults with type 2 diabetes regardless of BMI, provided the clinical indication for glucose lowering is met [91]. Thus, overweight individuals without obesity-related comorbidities generally do not meet current regulatory criteria for anti-obesity pharmacotherapy. People with lower BMI still manage to get on GLP-1 receptor agonist treatment even when they do not qualify; they still lose a substantial amount of body weight, although the magnitude tends to be smaller [92]. It is also important to note that GLP-1/GIP receptor agonists primarily maintain glycemic control while treatment continues. They rarely produce durable remission of type 2 diabetes after discontinuation, as illustrated in the semaglutide (Wegovy) STEP-1 extension study or tirzepatide’s SURPASS trials [93,94].

## 7. Gastrointestinal (GI) Effects of GLP-1RAs or GLP-1-Based MRAs

Gastrointestinal (GI) effects, including nausea, vomiting, diarrhea, and constipation, are among the most frequently reported adverse events for patients receiving GLP-1 receptor agonist (GLP-1RA) therapy (Table 2). The prevalence and duration of these effects vary among GLP-1RA classes, with short-acting agents causing more nausea and vomiting, and long-acting agents more often causing diarrhea [95]. The mechanism appears to involve both central nervous system and delayed gastric emptying effects [96]. Dose escalation appears to reduce GI-related discontinuation by making symptoms more tolerable during titration. Even so, some patients still stop treatment because of nausea, vomiting, or diarrhea, especially early in therapy. Importantly, GI side effects do not compromise the HbA1c- or weight-lowering efficacy of GLP-1RAs.

Most GI adverse effects arise during the dose-escalation period and tend to subside once a maintenance dose is reached, suggesting physiological adaptation [97]. Higher doses typically result in more effective weight loss but are associated with greater risk of nausea [98]; however, those with the most severe GI symptoms are not always those who lose the most weight, as weight loss is mediated primarily by appetite suppression. Thus, a balance must be struck between dose and tolerability [99].

GLP-1RAs have also been associated with rare cases of clinically significant gastroparesis, bowel obstruction or ileus, pancreatitis, and gallbladder or biliary disease. The strength of evidence and likely mechanisms differ among these events [100]. Delayed gastric emptying is a direct pharmacological effect of GLP-1 receptor activation, although the frequency with which it progresses to persistent or clinically significant gastroparesis remains uncertain [101]. Causal links with bowel obstruction and pancreatitis are less well established, whereas the increased risk of gallbladder and biliary disease is better supported and may reflect both impaired gallbladder motility and the indirect effects of substantial or rapid weight loss.

Although GI side effects are commonly linked to GLP-1RA therapy, treatment-associated changes in eating patterns, such as reduced meal size, altered food choices, and rapid reductions in caloric intake, may also influence their severity. Gradual dose escalation, consumption of smaller meals, avoidance of high-fat foods, and slower adjustment of food intake can help minimize discomfort and improve tolerance [99]. Dietary counseling may also be useful when therapy is discontinued, because abrupt resumption of larger meals may aggravate GI discomfort during the transition. Effective dietary management is therefore important for adherence and quality of life during both initiation and discontinuation of GLP-1RA therapy.

Notably, a recent phase 2 study of NG101, an oral, peripherally acting dopamine D_2_-receptor antagonist, demonstrated a ~40% reduction in nausea and a ~67% reduction in vomiting in patients receiving semaglutide, suggesting a promising approach to improving the tolerability of GLP-1 therapy and potentially enhancing long-term adherence [102].

## 8. GLP-1RA’s Effects on Muscle and Bone Density

Skeletal muscle mass and bone density are regulated by mechanical loading, the osteogenic and myogenic stimulus generated by forces exerted on the skeleton [103]. During the acute phase of weight loss, the reduction in mechanical strain diminishes this stimulus, leading to loss of lean muscle mass. However, clinical data on the extent of lean mass loss by different GLP-1RAs are mixed. The extent of lean muscle mass loss is confounded by a variety of factors, including different body-composition metrics (e.g., appendicular lean mass, skeletal muscle volume), tests that measure muscle strength and physical performance, and different ways to measure musculoskeletal health (e.g., DXA, MRI). Some trials have reported that up to 40% of total weight loss in patients treated with semaglutide is attributable to lean mass, while studies with tirzepatide have reported approximately 25% [13,104]. In contrast, other investigations using semaglutide or tirzepatide have observed lean mass losses closer to 15% of total weight loss [105]. These differences in body composition also warrant scrutiny in elderly populations and individuals with sarcopenic obesity, where compounded muscle loss directly accelerates frailty, increases fall risk, and reduces functional independence [106]. With the differences in imaging modality, patient population, trial comparators, and degree of weight loss, many meta-analyses have been conducted to elucidate these variables [104,107,108].

Although the exact magnitude varies across studies, incorporating lifestyle interventions, particularly resistance training, alongside GLP-1RA therapy remains the clinical standard for mitigating lean mass loss. Novel pharmacological therapies targeting the myostatin/activin pathway are currently under investigation to preserve muscle mass (see Section: Preserving muscle during weight loss and regaining muscle during weight rebound).

GLP-1 treatments have mixed effects on bone density. Rapid weight loss often causes some bone loss by reducing mechanical load and nutrient intake. However, GLP-1RA use has not been linked to an increased risk of fracture in patients with type 2 diabetes. In animal models, exenatide and liraglutide have even demonstrated potential bone-protective effects by decreasing osteoclast formation and promoting osteoblast activity, thereby enhancing bone formation, potentially offsetting bone loss from reduced mechanical load [109,110]. Despite these encouraging findings, human studies on bone strength have yielded inconsistent results, with some showing improvement, others no significant effect, and some reporting deterioration [111].

## 9. Neuropsychiatric Effects of GLP-1RAs

Beyond their well-established effects on appetite, GLP-1RAs increasingly appear to modulate central reward processing and motivated behaviors [16]. GLP-1 receptors are expressed in mesolimbic reward circuits, including the ventral tegmental area (VTA), nucleus accumbens, and other regions involved in hedonic feeding and reinforcement [16]. Clinical studies and preclinical models suggest that GLP-1RAs not only reduce caloric intake but may also alter food preferences, particularly by decreasing cravings for highly palatable, energy-dense foods rich in sugar and fat [112]. Emerging evidence further suggests reductions in alcohol consumption, nicotine use, and other compulsive or addictive behaviors in some individuals, although these observations are largely derived from retrospective analyses, observational studies, and animal experiments, and prospective randomized trials remain limited [112].

The long-term safety profile of GLP-1RAs has raised concerns regarding potential neuropsychiatric effects in early post-market reports. Some observational evidence has suggested a correlation between GLP-1 use and depressive disorders, while other population studies, including those focused on older adults, demonstrated no significant difference in the incidence of clinical depression [113,114,115]. Additionally, in a comparator, new user cohort study of T2D patients on GLP-1RAs, no elevated risk of suicidal ideation, self-harm, or suicide was found compared to patients on DPP-4 or SGLT-2 inhibitors [116]. To date, neither the U.S. Food and Drug Administration (FDA) nor the European Medicines Agency (EMA) has concluded that available evidence supports a causal association, although both continue to monitor post-marketing safety data.

**Table 2:** Safety profile of GLP-1 agonists.

GLP-1 Agonist	Common Treatment-Emergent Adverse Events (>5% of Patients in Study)	Labeled Warnings and Precautions	Theoretical Risks and Trial Signals
Dulaglutide [27,28]	- Nausea - Vomiting - Diarrhea - Dyspepsia - Constipation - Decreased appetite - Flatulence	- Pancreatitis - Gallbladder disorders - Hypoglycemia	- Thyroid carcinoma
Liraglutide [25,26]	- Nausea - Vomiting - Diarrhea - Dyspepsia - Constipation - Decreased appetite - Bronchitis - Nasopharyngitis - Upper respiratory tract infections - Headache - Abdominal pain - Back pain - Decreased appetite - Fatigue	- Pancreatitis - Gallbladder disorders - Hypoglycemia	- Thyroid carcinoma
Semaglutide [12,30,31]	- Nausea - Vomiting - Diarrhea - Dyspepsia - Constipation - Decreased appetite - Upper respiratory tract infection - Nasopharyngitis - Headache - Abdominal pain - Eructation	- Pancreatitis - Gallbladder disorders - Hypoglycemia - Diabetic retinopathy - Allergic reactions - Acute renal failure	- Benign and malignant neoplasms - Psychiatric disorders - Cardiovascular disorders - Hepatic disorders
Exenatide [24]	- Nausea - Vomiting - Diarrhea - Upper respiratory tract infections - Headache - Dizziness - Injection site erythema	- Pancreatitis - Hypoglycemia - Increase in heart rate	
Lixisenatide [29]	- Nausea - Vomiting - Diarrhea	- Pancreatitis - Hypoglycemia - Allergic reactions	
**GLP-1 Dual Agonist**			
Tirzepatide [13,32,33,34]	- Nausea - Diarrhea - Constipation - Dyspepsia - Vomiting - Decreased appetite - Headache - Abdominal pain - Dizziness - Eructation - Alopecia - Nasopharyngitis - Fatigue - Gastroesophageal reflux disease	- Pancreatitis - Major adverse cardiovascular events - Gallbladder disorders - Renal events - Hypoglycemia - Injection site reaction	- Psychiatric disorders
Mazdutide [35]	- Nausea - Diarrhea - Vomiting - Decreased appetite - Upper respiratory tract infection - Urinary tract infection - Hyperuricemia - Abdominal distention	- Pancreatitis - Gallbladder disorders - Hypoglycemia - Injection site reaction - Allergic reaction - Sinus tachycardia	
**GLP-1 Triagonist**			
Retatrutide [36,37]	- Nausea - Diarrhea - Constipation - Vomiting - Decreased appetite - Headache - Lipase increase - Urinary tract infection	- Hypoglycemia - Pancreatitis - Acute renal events	- Treatment- emergent anti-drug antibodies - Supraventricular arrhythmias and cardiac conduction disorders - Injection site reactions - Hepatic or biliary disorders - Hyperesthesia and related adverse events - Major adverse cardiovascular events

## 10. Current Directions in the Development of Next-Generation GLP-1RA Therapies

1. Insights from Eli Lilly and Novo Nordisk’s anti-obesity pipelines:

Novo Nordisk and Eli Lilly are two major companies in the field of anti-obesity medicine. Both have invested decades of effort into engineering incretin-based drugs, most notably GLP-1 receptor agonists and their multi-receptor derivatives, which remain the most effective pharmacological tools for weight loss (Table 3). Their ongoing clinical trials and development programs currently serve as the primary clinical and regulatory benchmarks in the incretin landscape, providing the standard against which emerging pharmacological interventions are evaluated.

**Table 3:** Novo Nordisk’s and Eli Lilly’s current pipelines for obesity.

Target Receptor(s)	Drug Candidate	Formulation	Development Phase	Regulatory Status
**Novo Nordisk**				
GLP-1R	Semaglutide (7.2 mg)	Subcutaneous	Phase 3	Completed (FDA Approved)
GLP-1R/Amylin	CagriSema	Subcutaneous	Phase 3	Completed (FDA Submission Pending)
GLP-1R/Amylin	Zenagamtide (Amycretin)	Subcutaneous	Phase 3	Ongoing
FGF21	Efruxifermin	Subcutaneous	Phase 3	Ongoing
Amylin	Cagrilintide	Subcutaneous	Phase 3	Ongoing
GLP-1R/Amylin	Zenagamtide (Amycretin)	Oral	Phase 3	To be initiated
CB1R Antagonist	Monlunabant	Oral	Phase 2	Ongoing
GLP-1R/GIPR/GCGR	UBT251	Subcutaneous	Phase 2	Ongoing (Phase 1b/2a Global)
GLP-1R/GIPR/Amylin	Triple	Subcutaneous	Phase 2	Ongoing
Amylin	Amylin 355	Subcutaneous	Phase 1	Ongoing
Amylin	Amylin 1213	Subcutaneous	Phase 1	Ongoing
ACSL5 Antagonist	LX9851	Oral	Phase 1	Ongoing
GLP-1R	N/A	Subcutaneous	Phase 1	Ongoing
SLC25A5	N/A	N/A	Phase 1	Ongoing
**Eli Lilly**				
GLP-1R	Orforglipron	Oral	Phase 3	Completed (FDA Approved)
GLP-1R/GIPR/GCGR	Retatrutide	Subcutaneous	Phase 3	Ongoing
GLP-1R/GCGR	Mazdutide	Subcutaneous	Phase 2	Ongoing
Myostatin Antagonist	Bimagrumab	Subcutaneous	Phase 2	Ongoing
Amylin	Eloralintide	Subcutaneous	Phase 2	Ongoing
GLP-1R (NPA)	Naperiglipron	Oral	Phase 2	Ongoing
PYY	Nisotirostide	Subcutaneous	Phase 2	Ongoing
GIPR	Macupatide	Subcutaneous	Phase 2	Ongoing
GLP-1R/GIPR	Brenipatide	Subcutaneous	Phase 1	To be initiated

Note: Pipeline information, clinical trial phases, and regulatory statuses are current as of July 2026. Data was sourced from ClinicalTrials.gov, official Novo Nordisk pipeline releases, and official Eli Lilly pipeline releases. Phase 1 trials are no longer reported publicly on official Eli Lilly pipeline releases.

Novo Nordisk’s current strategy appears to be a multi-pronged approach for its established medications, prioritizing diverse treatment options, patient convenience, and market expansion. Their decision to aggressively pursue amylin analogs, including combinations with semaglutide (CagriSema), as well as novel unimolecular dual and triple agonists, is clear based on the release of their upcoming pipeline. One promising candidate is amycretin, a unimolecular GLP-1/amylin dual agonist that is currently being investigated as a once-weekly subcutaneous injectable and a once-daily oral pill. While CagriSema represents a significant step forward in incretin/amylin therapy, amycretin has the potential to be a superior successor, offering more potent weight loss in a shorter timeframe and an attractive oral formulation. After evaluation of several doses of amycretin (1.25 mg vs. 5 mg vs. 20 mg vs. 60 mg) in phase 1b/2a trials, 20 mg appeared to have the greatest balance between adverse events and efficacy, achieving −22% weight loss in just 36 weeks compared to CagriSema’s −20.4% weight loss in 68 weeks [117,118]. Although the 60 mg group achieved −24.3% weight loss in 36 weeks, 35% of patients withdrew from the study due to adverse events [117].

Eli Lilly, on the other hand, seems to prioritize a multi-hormone approach as seen through their diverse portfolio, while also emphasizing combinations of GLP-1 with GIP and/or glucagon. Based on their 2026 Q1 report, most of their late-stage clinical trials are exploring the use of tirzepatide, retatrutide, and orforglipron in metabolic conditions other than obesity. While they are also exploring the amylin strategy with eloralintide, it is interesting that Lilly is exploring a Peptide YY (PYY) agonist, given a track record of modest efficacy.

2. Other Multi-Functional Molecules (MRAs and Combinations):

While Novo Nordisk re-established weight loss as a legitimate field for drug development with liraglutide after decades of failures and notable historical setbacks, Eli Lilly has since taken the lead with the FDA approval of the first GLP-1R/GIPR dual agonist, tirzepatide.

In this context, it is important to examine Novo Nordisk’s most advanced contender, CagriSema, which combines an amylin analog with a GLP-1 receptor agonist. Notably, CagriSema is not a dual agonist in the strict pharmacological sense but rather a co-formulation of two separate drugs [119]. Amylin is a hormone co-secreted with insulin in response to nutrient intake and slows gastric emptying and induces satiety. Cagrilintide is a long-acting amylin analog that is being studied in conjunction with semaglutide under the investigational name CagriSema [119]. A 32-week phase 2 trial showed that this combination of cagrilintide with semaglutide resulted in greater weight loss (−15.6%) compared to semaglutide (−5.1%) or cagrilintide (−8.1%) alone. HbA1c levels after 32 weeks were −2.2% for CagriSema, −1.8% for semaglutide, and −0.9% for cagrilintide [119]. Newly released phase 3 REDEFINE program results further validate CagriSema’s potential. In REDEFINE-1, which focused on adults who were overweight or obese without diabetes, CagriSema achieved a mean weight loss of 22.7% after 68 weeks, rivaling the efficacy seen with tirzepatide [118]. In REDEFINE-2, which included patients with type 2 diabetes and obesity, the combination also achieved a meaningful weight-loss reduction of −13.7%, suggesting its promise across metabolic phenotypes [120]. A total of 75.9% of participants eventually reached the final dosage of 2.4 mg cagrilintide and 2.4 mg semaglutide [120].

Viking Therapeutics is also actively developing a GLP-1R/GIPR dual agonist (VK 2735) as both a weekly subcutaneous injection and as an oral tablet. After 13 weeks, phase 2 results of the subcutaneous injection formulation indicated an average weight loss of 9.2 kg, 10.7 kg, 13.3 kg, and 14.6 kg in the 2.5 mg, 5 mg, 10 mg, and 15 mg groups, respectively [121]. In comparison, patients achieved a mean weight loss of 2.1 kg, 4.4 kg, and 6.2 kg in the 5 mg, 10 mg, and 15 mg groups, respectively, with tirzepatide after 26 weeks [122]. The safety profile closely mirrors that of tirzepatide, with nausea and other gastrointestinal events being the most common adverse effects [121]. These findings position VK2735 as a strong competitor to tirzepatide in terms of both efficacy and safety [121].

There are also efforts to develop a peptide based on genetic variants. Amgen and decODE genetics have looked at variants associated with low BMI in human data and found that individuals with variants that reduced GIP activity were found to have lower BMIs [123]. This led to the development of MariTide (AMG133), a molecule that activates the GLP-1 receptor and simultaneously inhibits GIPR [123]. The molecule’s design as a monoclonal antibody targeting the GIP receptor allows for less frequent administration, offering an advantage over traditional peptide therapies. In male obese mice and cynomolgus monkeys, AMG133 demonstrated a dose-dependent decrease in body weight and improved metabolic markers [123]. In a phase 1, randomized, double-blind, placebo-controlled clinical study in participants with obesity, a 140 mg dosage of AMG133 led to a 7.4% decrease in body weight after 78 days, while a dosage of 420 mg led to a 14.5% decrease in body weight after 85 days [123]. In comparison, the placebo group had a mean decrease in body weight of just 1.5% after 85 days. Phase 2 clinical trial results confirmed and extended these findings. Among participants with obesity but without type 2 diabetes, AMG133 led to a mean weight loss of 20% after 52 weeks, using a dose escalation up to a 420 mg monthly dose [124]. In participants with both obesity and type 2 diabetes, weight loss reached 17% across dosing arms (140 mg, 280 mg, 420 mg monthly) [124]. Notably, weight loss did not plateau after 52 weeks, suggesting potential for continued benefit with longer treatment durations [124].

Except for VK2735, which closely resembles tirzepatide yet appears to deliver superior weight-loss efficacy, candidates such as CagriSema and MariTide underperform tirzepatide, let alone Lilly’s triagonist retatrutide, raising doubts about their widespread clinical use even if approved [120,124]. While Novo Nordisk and Eli Lilly continue to focus on crowded receptor spaces, many smaller biotechnology and pharmaceutical companies are pursuing less canonical targets such as GLP-2, fibroblast growth factor 21 (FGF21), melanocortin-4 receptor (MC4R), insulin growth factor-1 (IGF-1), calcitonin, and peroxisome proliferator-activated receptors (PPARs) within unimolecular MRAs. These novel candidates will have to not only demonstrate niche clinical utility but also meet the high efficacy and safety thresholds established by larger pharmaceutical companies. As of mid-2026, there are estimated to be over 100 GLP-1-centered pipeline assets in global clinical development for obesity and type 2 diabetes; the table below covers all major targets but only highlights a subset of ongoing clinical trials in this area (Table 4). A key open question remains: which therapeutic strategy—GLP-1/GIP agonism, GLP-1/GIP antagonism, or GLP-1/amylin synergy—will ultimately deliver the optimal balance of efficacy, safety, and patient adherence?

**Table 4:** Multi-receptor agonists in development.

Name	Type	Target Receptor	Company	Status	Clinical Trial
Survodutide	Dual	GLP-1/GCG	Boehringer Ingelheim	Phase 3	NCT06077864
VK2735	Dual	GLP-1/GIP	Viking Therapeutics	Phase 3	NCT07104500
AMG133	Dual	GLP-1/GIP antagonist	Amgen	Phase 3	NCT06858878
BGM0504	Dual	GLP-1/GIP	BrightGene	Phase 3	NCT06704581
Amycretin	Dual	GLP-1/amylin	Novo Nordisk	Phase 2	NCT05369390
Efinopegdutide	Dual	GLP-1/GCG	Merck/Hanmi	Phase 2	NCT05877547
Pemvidutide	Dual	GLP-1/GCG	Altimmune/Spitfire	Phase 2	NCT06987513
Dapiglutide	Dual	GLP-1/GLP-2	Zealand Pharma	Phase 2	NCT05788601
NNC0165-1875 + Semaglutide	Dual *	GLP-1/PYY	Novo Nordisk	Phase 2	NCT04969939
NNC0165-1562 + Semaglutide	Dual *	GLP-1/PYY	Novo Nordisk	Phase 1	NCT03574584
MET-233i + MET-097i	Dual *	GLP-1 + amylin	Metsera/Pfizer	Phase 1	NCT06924320
SCO-094	Dual	GLP-1/GIP	Scohia Pharma	Phase 1	Japan: JRCT1080225227
CT388	Dual	GLP-1/GIP	Carmot Therapeutics	Phase 1	NCT06628362
UBT251	Triple	GLP-1/GIP/GCG	United Biotechnology/Novo Nordisk	Phase 2	NCT07177469
MWN-109	Triple	GLP-1/GIP/GCG	Shanghai Minwei Biotechnology	Phase 2	NCT06859853
Efocipegtrutide	Triple	GLP-1/GIP/GCG	Hanmi	Phase 2	NCT04505436
DR-10624	Triple	GLP-1/GCG/FGF-21	Zhejiang Doer Biologics	Phase 2	NCT07024212
Bremelanotide + Tirzepatide	Triple *	GLP-1/GIP/MC4R	Palatin Technologies	Phase 2	NCT06565611
Bioglutide	Tetra	GLP-1/GIP/GCG/IGF-1	Biomed Industries	Phase 2	NCT06564753
PTT-A	Tetra	GLP-1/GIP/Amylin/Calcitonin	Pep2Tango Therapeutics	Preclinical [125]	
N/A	Tetra	GLP-1/GIP/GCG/PYY	Tufts University	Preclinical [126]	
Lanifibranor/GLP-1/GIP	Penta	GLP-1/GIP/Pan-PPAR	German Center for Diabetes Research	Preclinical [127]	

*: not a dual or triagonist in the strict pharmacological sense but rather a co-formulation of separate single-receptor agonists.

3. Oral GLP-1 Receptor Agonists

Most GLP-1 receptor agonists currently available are peptide-based drugs, such as exenatide, liraglutide, and semaglutide, which are administered via injection due to their large molecular size and poor oral bioavailability. While injectable GLP-1 agonists have been proven effective, oral GLP-1 agonists would increase adherence and convenience.

Pfizer developed an oral small-molecule GLP-1 agonist, danuglipron, as an alternative to injectables. Danuglipron demonstrated an in vitro signaling profile similar to that of GLP-1, potentiated glucose-stimulated insulin release, decreased the rate of food intake in monkeys, and demonstrated oral bioavailability in early human trials [128]. However, Pfizer has discontinued the development of danuglipron due to safety concerns from drug-induced liver injury and liver enzyme elevations [129].

Orforglipron, developed by Eli Lilly, is an FDA-approved oral small-molecule GLP-1R agonist for adults with obesity or overweight with weight-related medical problems. Mechanistically, it preferentially activates G protein/cAMP signaling with reduced β-arrestin recruitment and receptor internalization [130]. In phase 3 trials, orforglipron achieved meaningful reductions in body weight of −7.5%, −8.4%, and −11.2% in the 6 mg, 12 mg, and 36 mg groups, respectively, after 72 weeks [131]. In the ACHIEVE-3 head-to-head trials, orforglipron was found to be superior to oral semaglutide for weight loss after 52 weeks, with the 36 mg dose achieving a 9.2% reduction in body weight compared to 5.3% with 14 mg semaglutide [132]. However, previous clinical trials with higher doses of oral semaglutide, 25 mg and 50 mg, showed significantly greater weight loss than orforglipron at −13.6% (64 weeks) and −15.1% (68 weeks), respectively [31,133].

In contrast, Novo Nordisk has taken a different approach to enable the oral administration of its small peptide drug, semaglutide. SNAC (sodium N-(8-[2-hydroxybenzoyl]amino)caprylate) was identified in the 1990s as the lead permeation enhancer within Emisphere Technologies’ Eligen^®^ carrier technology. Emisphere researchers demonstrated that SNAC functions as a localized buffer, temporarily neutralizing stomach pH to shield the GLP-1 from enzymatic breakdown [134]. SNAC also fluidizes gastric cell membranes, increasing their permeability to allow the peptide to pass through [134]. The compound gained broad attention when Novo Nordisk incorporated it into the oral formulation of semaglutide (Rybelsus^®^), the first clinically approved peptide tablet. This success established SNAC as a practical excipient for oral peptide delivery, with subsequent reviews highlighting its role in facilitating gastric absorption of semaglutide [135]. Other chemical permeation enhancers, including sodium caprate, sodium caprylate, tetradecyl maltoside, sodium cholate/deoxycholate, and lauroyl carnitine, have been investigated to improve oral peptide delivery. Although these enhancers remain dominant, the field is increasingly exploring structural stabilization strategies for the peptides themselves. By synthesizing cyclic peptides, integrating heavy fluorination, or engineering intrinsic hydrocarbon staples, these molecules can achieve high oral bioavailability (20% to 40% in preclinical models) without requiring formulation enhancers [136].

4. New Receptor Engagement Format

In recent years, the concept of biased agonism has emerged as an important focus in GLP-1R pharmacology [137,138,139]. Structural or chemical modifications of GLP-1R agonists can differentially engage G protein- versus β-arrestin-mediated signaling pathways. Agonists that preferentially activate Gs signaling tend to maximize metabolic benefits, such as enhanced insulin secretion, appetite suppression, and weight reduction, while minimizing tolerance and gastrointestinal side effects that are often associated with β-arrestin-driven receptor desensitization and internalization [68,138]. By contrast, β-arrestin-biased agonists may offer unique advantages in cardiovascular or neuroprotective contexts but could also increase the risk of adverse effects [62,68,138,140]. For example, ecnoglutide is a receptor-biased agonist for GLP-1 that has been shown to have significant effects on blood glucose, HbA1c levels, and body-weight reductions [141]. In phase 2 trials, the HbA1c-lowering effect of 1.2 mg ecnoglutide was found to be more effective than 1.0 mg semaglutide and comparable to 15 mg weekly tirzepatide [142]. Its alanine to valine substitution at position 8 biases GLP-1 receptor signaling by favoring cAMP induction over β-arrestin recruitment, prolongs insulin secretion and delays the internalization of the GLP-1 receptor [141].

Building on the concept of biased signaling, differences in ligand type, particularly peptides versus small molecules, can also shape GLP-1 signaling bias. GLP-1R’s orthosteric site is structurally optimized to bind long peptide hormones such as GLP-1, exendin-4, and oxyntomodulin. These peptides can engage both the extracellular domain (ECD) and the transmembrane (TM) core through a two-domain binding mode [63,143,144], but this does not necessarily produce balanced signaling, since peptide agonists themselves can display distinct patterns of G-protein versus β-arrestin engagement and trafficking [145]. Small molecules, by contrast, make fewer contacts with GLP-1R and often stabilize a more limited set of receptor conformations, so they usually bind allosteric or partial-orthosteric pockets within the TM bundle [138]. Although small molecules can fully activate GLP-1R in at least some signaling dimensions (notably cAMP), they typically show biased and non-identical signaling profiles compared with peptide agonists and may not be “fully activating” in a global, pathway-agnostic sense [146].

Another emerging design is the antibody-peptide conjugate (APC). For example, Twist Bioscience’s TB59-2 is engineered by fusing a GLP-1 peptide to a specific, non-functional anti-GLP-1R antibody (TB01-2) [147]. Once the antibody anchors itself to the receptor, the GLP-1 peptide attached to it is held in extremely close proximity to the receptor’s activation site (orthosteric site). The peptide binds to the site, activates it, and then dissociates (“off”). Because it is tethered, it cannot diffuse away. The high local concentration forces it to immediately re-bind (“bind”) and activate the receptor again. This cycle repeats, allowing a single antibody–peptide complex to activate the receptor multiple times (sustained signaling) without the peptide being degraded or lost. The antibody creates a long-lasting depot (weeks) because antibodies naturally persist in the blood for a long time. The “forced proximity” ensures the receptor is constantly stimulated, mimicking a continuous infusion of the drug.

5. Preserving muscle during weight loss

One of the primary challenges of weight-loss therapies is preserving skeletal muscle mass during weight loss. Skeletal muscle is a key regulator of glucose homeostasis and total-body energy expenditure; more muscle usually correlates with better glucose homeostasis and higher energy expenditure.

During weight loss and energy restriction, resistance training has consistently been identified as the primary non-pharmacologic modality for maintaining or increasing muscle mass. In contrast to aerobic training, the mechanical tension provided by resistance training activates the mTORC1 pathway, stimulating protein synthesis even in a caloric deficit [148,149]. In a twelve-week comparative study, subjects on an 800-calorie liquid diet who underwent aerobic training achieved greater total weight loss (~19.4%) but experienced significant loss of lean body mass, while the resistance training group had moderate weight loss (~14.7%) with no significant loss of lean body mass [150].

The timing of resistance training may be particularly important for optimizing long-term muscle preservation. Evidence from muscle-memory studies indicates that resistance training induces myonuclear accretion early in the hypertrophic process and that these myonuclei can persist during detraining, enabling more rapid muscle regrowth later in life [148,151]. Early resistance training with GLP-1RAs may optimize myonuclear accumulation and thereby preserve the ability to maintain or regain skeletal muscle mass during later stages of treatment or during weight rebound. Additionally, skeletal muscle experiences anabolic resistance during caloric restriction, whereby the normal protein synthetic response to dietary protein and mechanical loading is blunted [152,153]. This resistance can be partially overcome through higher protein intakes and aggressive resistance training protocols, though the protective effect is modest [152,153].

However, to overcome the ceiling of non-pharmacological interventions, pharmaceutical agents have been developed to inhibit the myostatin (GDF8) pathway, a key negative regulator of muscle growth and development [154]. Myostatin exerts its effects by binding to activin type II A and B (ActRII) receptors, subsequently recruiting activin-like kinase (ALK) 4/5, which leads to the phosphorylation of transcription factors Smad 2/3; Smad2/3 downregulate MyoD, inhibiting myogenic differentiation and driving muscle atrophy [154].

Bimagrumab, a monoclonal antibody that inhibits ActRII receptors and thereby blocks myostatin signaling, has been shown to effectively decrease fat mass, increase lean mass, and improve insulin sensitivity and HbA1c levels [155]. Originally investigated for pathological muscle loss and weakness in sporadic inclusion body myositis, bimagrumab has now shifted into the metabolic landscape as an adjunct to incretin therapies. In a recent phase 2b study (BELIEVE), bimagrumab combined with semaglutide produced a 22.1% reduction in body weight, significantly greater than either drug alone (15.7% with semaglutide, 10.8% with bimagrumab) (NCT05616013) [156]. Notably, nearly all the weight loss with bimagrumab was derived from fat mass: 100% when bimagrumab was used alone and 92.8% when combined with semaglutide, with lean mass preserved or increased. The safety and efficacy of bimagrumab in conjunction with tirzepatide (NCT06643728) are currently being evaluated in clinical trials.

Other myostatin inhibitors of interest include apitegromab (Scholar Rock) and trevogrumab (Regeneron). Apitegromab differentiates itself by binding to human promyostatin and inactive myostatin rather than targeting the ActRII receptor [157]. While primarily studied for spinal muscular atrophy, it has shown promise as an adjunct in obesity therapy. The phase 2 EMBRAZE trial, which evaluated apitegromab with tirzepatide, demonstrated that 85.3% of the total mass loss was due to fat mass loss compared to 69.5% with tirzepatide alone (NCT06445075) [158]. Trevogrumab is another myostatin monoclonal antibody that binds to and neutralizes myostatin. It has been tested alongside semaglutide in the phase 2 COURAGE study. According to preliminary company-reported data, trevogrumab with semaglutide preserved 51.3% of lean muscle mass that would be lost during semaglutide treatment (NCT06299098) [159].

In the same TGFβ family and working on the same ActIIA and ActIIB receptors but engaging distinct Alk receptors, Activin A and Activin E are also targets of pharmaceutical intervention [160,161,162]. Activin A, encoded by INHBA, binds ActRIIA/B, which then recruits ALK4 to activate SMAD2/3 and suppress MyoD-driven myogenic differentiation. Activin A–ActRII–SMAD2/3 signaling overlaps with myostatin in skeletal muscle to inhibit myogenesis, while in adipose tissue, Activin A is upregulated in obesity and promotes expansion and self-renewal of adipocyte progenitors [163,164]. Garetosmab is an experimental, fully human monoclonal antibody designed to bind to activin A [165]. In preliminary company-reported data, Regeneron demonstrated that combining semaglutide with activin A neutralization (garetosmab) and myostatin neutralization (trevogrumab) preserved 80.9% of the lean mass that would otherwise be lost during standard semaglutide treatment (NCT06299098) [159].

Despite these promising effects on body composition, the functional impact of myostatin inhibition remains under scrutiny. Clinical trials in neuromuscular disorders such as Duchenne muscular dystrophy (DMD) and inclusion body myositis have shown that while myostatin-targeting drugs can modestly increase muscle size, this has not consistently translated into improved strength or physical function [166]. Future trials combining myostatin inhibitors with incretin therapies should include physical performance tests or biomarker measurements to assess muscle function. One promising strategy to address this limitation involves Biolinq’s continuous protein biosensor, a minimally invasive tool for tracking optimal protein intake and muscle mass loss during treatment with GLP-1R agonists [167].

Additionally, treatment-related muscle spasms are the biggest concern for drugs targeting this pathway. For example, they were more frequent in the anti-GDF8 + anti-ActA combination groups compared with placebo, with a difference in frequency greater than 40% in Regeneron’s phase 1 clinical trial [168]. A high incidence of muscle spasms was seen again in their phase 2 COURAGE Trial, which adds semaglutide to the treatment. The most common adverse event was muscle spasm, occurring in 4.6% with semaglutide alone, 6.1% with semaglutide + trevogrumab 200 mg, and 9.3% with semaglutide + trevogrumab 400 mg, compared with 40.9% with the triple combination, leading to a 30.9% treatment discontinuation in the triple combination group [169].

A newer pharmacological format, RNAi, may also improve body composition during weight loss by targeting liver-secreted proteins. RNAi therapies against Inhibin Subunit Beta E (INHBE) are in clinical development by Wave Life Sciences (WVE-007) and Arrowhead Pharmaceuticals (ARO-INHBE). INHBE encodes Activin E, a liver-derived hepatokine that signals via ActRIIA/B and ALK7 (ACVR1C) in white adipose tissue to activate SMAD2/3 and repress lipolysis, forming a liver–adipose feedback loop in which hepatic lipid load induces INHBE and circulating Activin E then restrains adipocyte fatty-acid efflux [170,171]. From a human-genetics standpoint, multi-ancestry exome and GWAS studies identify rare INHBE loss-of-function variants associated with lower waist-to-hip ratio (WHR) adjusted for BMI, reduced visceral fat, and protection from type 2 diabetes without loss of lean mass, while common variation at INHBA and related activin-pathway loci associates with WHR and regional fat distribution [172,173,174]. WVE-007 and ARO-INHBE use GalNAc-conjugated RNAi to silence hepatic INHBE, lower circulating Activin E, and disinhibit adipose tissue fat loss; early reports show meaningful visceral fat reduction with preserved or increased lean mass, and combinations with semaglutide or tirzepatide approximately double weight loss and drive a higher proportion of visceral fat loss compared to incretin monotherapy [171,175,176,177]. In parallel, ARO-ALK7 targets the ALK7 receptor in adipose tissue, blocking Activin E–ALK7–SMAD2/3 signaling at the receptor level and providing a complementary way to modulate the same hepatokine–adipocyte axis [175,178,179].

## 11. Rationale for Making Multi-Receptor Agonists

Unimolecular multi-receptor agonists (MRAs) that simultaneously target GLP-1, GIP, and glucagon receptors have demonstrated superior efficacy compared with GLP-1 monotherapy in improving metabolic control and promoting weight loss across multiple recent preclinical and clinical studies. As such, they represent a major direction in the development of next-generation incretin-based therapeutics by the pharmaceutical and biotechnology industries. Despite their apparent increased potency, the mechanisms underlying the enhanced efficacy of unimolecular MRAs remain incompletely understood, limiting rational optimization and therapeutic refinement.

**Figure 3:**
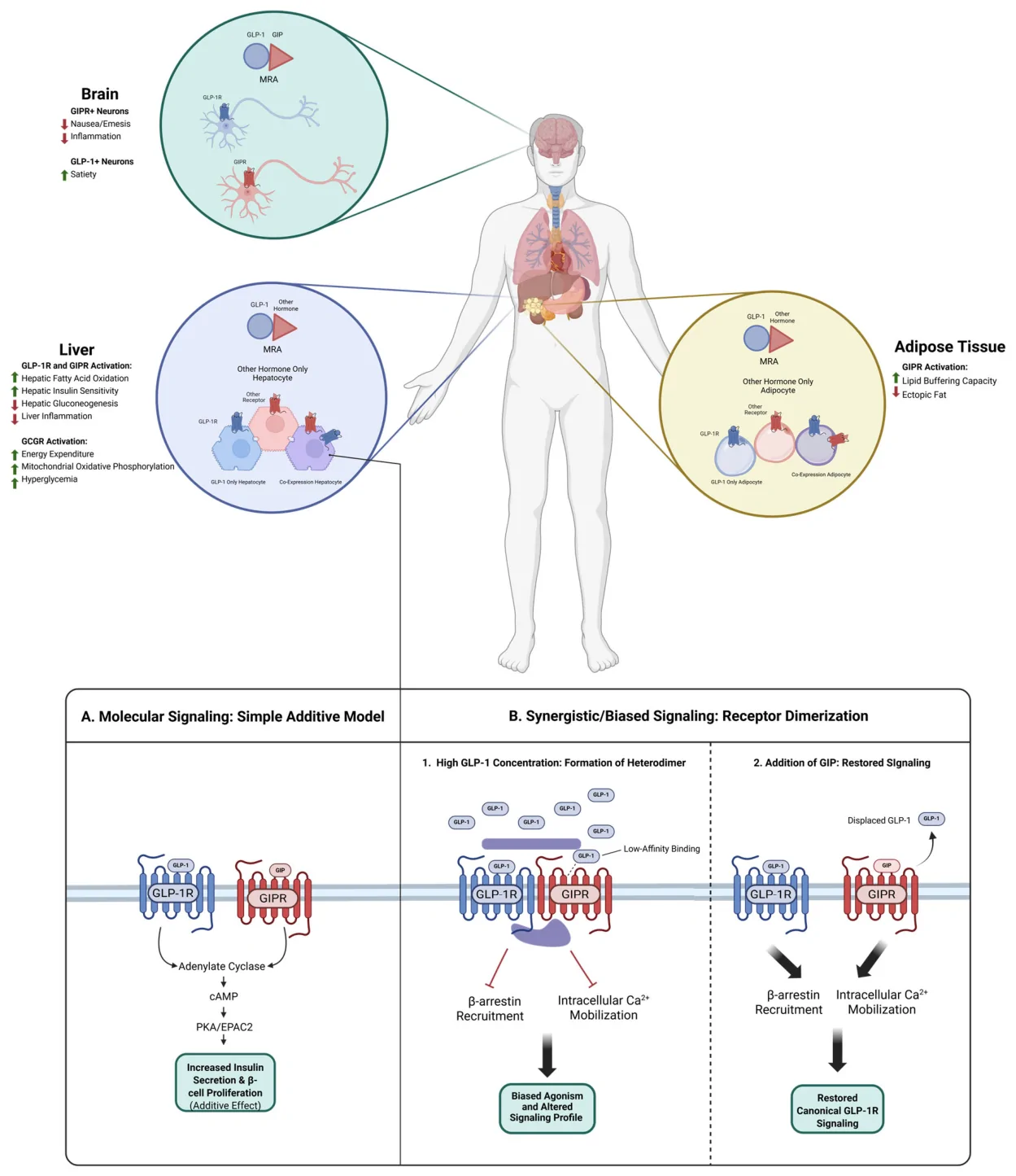
Tissue-specific actions and potential mechanisms of GLP-1 based multi-receptor agonists. (**A**) Activation of GLP-1R+ neurons in the hindbrain promotes satiety, while activation of GIPR+ neurons suppresses nausea and emesis. GCGR activation in the liver increases energy expenditure, mitochondrial oxidative phosphorylation, and fatty acid oxidation, although it requires GLP-1/GIP counter-regulation to manage hyperglycemia. GIPR activation in adipose tissue enhances lipid-buffering capacity and reduces ectopic fat, while contributing to anti-inflammatory effects. (**B**) Two mechanisms of multi-receptor agonists have been proposed based on the notion that MRAs typically bind to a single receptor at a time. In cells co-expressing receptors for GLP-1 and other metabolic hormones, simultaneous activation leads to additive production of cAMP via adenylate cyclase, stimulating the PKA/EPAC2 pathway and promoting the additive effect of insulin secretion and β-cell proliferation. The synergistic signaling mechanism promotes the formation of GLP-1R/GIPR heterodimers during high concentrations of GLP-1, resulting in a reduction in β-arrestin recruitment and intracellular calcium mobilization. The presence of GIP restores or alters signaling profiles through low-affinity binding.

Here, we outline several current hypotheses on how these multi-receptor agonists achieve their superior therapeutic effects and discuss the rationale for including additional receptor-engagement formats beyond GLP-1R (Figure 3).

1. Do MRAs Really Work Better Than the Combination of Individual Single-Receptor Agonists?

Most peptide MRAs bind to a single receptor at any given time, but they may engage different receptors on the same cell with multiple MRAs, thereby exerting additive metabolic effects by simultaneously stimulating multiple pathways in the same cell. GLP-1R and GIPR both activate adenylate cyclase, resulting in increased intracellular cAMP, which promotes insulin secretion, β-cell proliferation, and anti-apoptotic effects through activation of PKA and EPAC2 [180].

Although it is commonly argued that unimolecular multi-receptor agonists provide greater metabolic benefits than single-receptor agonists, direct head-to-head trials are scarce, and dose-matched comparisons are non-existent. Accordingly, it is not possible to draw definitive conclusions about their superiority over an optimally dosed combination of individual agonists with matched receptor potency, especially since such matched comparators are seldom available and are unlikely to be studied in industry-sponsored clinical trials. GLP-1 agonists induce weight loss in a dose-dependent manner, indicating that the extent of GLP-1R engagement is a key determinant of therapeutic efficacy. Most studies have not yet reached a plateau in weight loss, as the tested doses have not fully saturated the receptor.

For example, one study reported that “addition of GIP activity into dual GLP-1 and GCG receptor agonism provides improved effects on weight loss and glycemic control” in diet-induced obese mice. However, their triagonist (SAR441255) demonstrated greater potency on human receptors than their dual GLP-1R/GCGR agonist [181,182]. Please note EC50 values on mouse receptors should be compared here, but such data were not available in the study. The bottom line here is that while a triagonist with higher intrinsic potency at GLP-1R may yield superior efficacy, this does not necessarily imply that the third receptor component contributes meaningfully to the observed effects. Ideally, to demonstrate that GIP receptor agonism contributes additional weight loss, clinical trials would include GLP-1 receptor agonists combined with varying doses of a GIP receptor agonist. However, such studies have not been conducted and are unlikely to be pursued due to business considerations in pharmaceutical trial design.

In conclusion, the rapid growth of MRAs seems driven not only by biology but also by practical and commercial factors. A single unimolecular drug is easier to develop, test, and market than multiple separate agents, and it allows one regulatory pathway and one product label instead of complex multi-arm combination trials.

2. Integrated Tissue Actions of Multi-Receptor Agonists

Metabolic benefits are achieved through coordinated metabolic actions across different tissues, such as adipose tissue, brain, and gastrointestinal tract. For small-molecule or peptide-based MRAs, which bind only one receptor at a time, receptor engagement can also reshape pharmacokinetics and tissue distribution in ways that indirectly modulate the activity of the other pharmacophore within the same construct. Binding to one receptor can drive internalization, recycling, or localized retention in specific tissues, altering where and for how long the remaining active component can act.

Importantly, it is not common for a single cell to express all the different receptors targeted by MRAs, and the tissue distribution of GLP-1R and GIPR shows substantial heterogeneity with divergent expression patterns across different cell types and organs [183]. Because small-molecule and peptide-based MRAs can only bind to one receptor at a time (they lack the molecular architecture for concurrent engagement of multiple receptors), their effects are rarely mediated through simultaneous activation of all target receptors within the same cell [184]. Instead, MRAs likely enhance metabolic efficacy by engaging complementary receptor populations within and across tissues—for example, distinct neuronal populations in the brain, overlapping but non-identical receptor pools in pancreatic islets, and peripheral targets such as GIPR-expressing adipocytes and GCGR-expressing hepatocytes—thereby generating a broader multi-tissue metabolic response than single-receptor agonists can provide [183].

These coordinated, tissue-specific actions are central to the glycemic and weight-loss effects of dual and triple agonists. Tirzepatide’s GIPR agonism, for example, has been shown to modulate metabolic activity in adipose tissue by regulating triglyceride uptake and lipolysis during the fed and fasted states [185]. Additionally, GIP may enhance the lipid-buffering capacity of white adipose tissue, thereby reducing ectopic fat accumulation in other tissues [186]. Beyond direct metabolic control, GIP may also play a role in inflammation [187]. GLP-1/GIPR co-agonists retain anti-inflammatory action even in brain-specific GLP-1-deficient mice, suggesting that GIP may exert peripheral immune-modulating actions through adipose and immune cell pathways.

The central nervous system (CNS) also plays a critical role in mediating the anorexigenic effects of multi-receptor agonists. GIP and GLP-1 receptors are expressed in brain regions involved in energy balance, satiety, and control of emesis, including the nucleus tractus solitarius, area postrema, and the hindbrain [188]. In pre-clinical models, GIPR-expressing neurons may function as local inhibitory circuits that suppress the nausea and vomiting associated with GLP-1R activation, without blunting the desired satiety and glucose-lowering effects [188]. This specific neural interaction is thought to contribute to the tolerability profile of tirzepatide, allowing for higher dosing relative to semaglutide.

In the case of retatrutide, the mechanism involves a synergistic interaction among the GLP-1, GIP, and GCG receptors. The addition of the GCG receptor activity appears to drive greater reductions in liver fat and body weight compared to mono- and dual agonists, with patients achieving 86% relative reduction in liver fat at 48 weeks [189,190]. Comparative data support this, suggesting that glucagon agonism provides liver-specific benefits beyond weight loss alone. For example, dual GLP-1/GCG agonists like efinopegdutide achieved greater liver fat reductions than semaglutide [191]. These effects appear to be distinct from those of FGF21, as retatrutide suppressed circulating FGF21 levels, indicating its efficacy is independent of FGF21 signaling [189].

3. Adding Glucagon Receptor Agonism for Increasing Energy Expenditure

Reducing food intake is a common first step in weight loss. However, such interventions often lead to a compensatory decrease in energy expenditure [192,193,194], thereby limiting further weight loss. To achieve sustained weight reduction, maintaining energy expenditure has become a key strategy in the development of anti-obesity therapies [195,196]. But the key question is where the extra energy is dissipated and at what safety cost. Systemic mitochondrial uncoupling, which dissipates metabolic energy as heat by bypassing ATP synthesis, can drive significant weight loss but poses prohibitive risks of hyperthermia, cardiac arrhythmias, and sudden death in humans, as illustrated by the historical use of 2,4-dinitrophenol [197]. Tissue-selective thermogenesis, particularly in brown and beige adipose tissue, has been an attractive strategy because UCP1-mediated uncoupling in adipocytes can markedly increase local energy dissipation without necessarily affecting other organs; however, robust and sustained activation of human brown fat has been difficult to achieve, and the quantitative contribution of beige fat thermogenesis to long-term weight loss remains uncertain [198,199]. Sympathomimetic and other adrenergic receptor agonists can similarly increase thermogenesis and lipolysis, but experience with earlier generations of these agents underscores the risk of cardiovascular and neuropsychiatric adverse effects [200], highlighting the safety issues inherent in broadly stimulating the sympathetic nervous system.

GCG, a 29-amino acid peptide processed and secreted by pancreatic α-cells, exerts its physiological function via its receptor GCGR. The site of action is the liver, consistent with the expression pattern of its receptor (GCGR) [201]. GCGR expression in the liver is more than 100-fold higher than in the kidney, the second-highest tissue [202]. Glucagon increases energy expenditure by stimulating mitochondrial oxidative phosphorylation, thereby increasing oxygen consumption and ATP production [203,204,205]. Despite these beneficial effects, the clinical utility of glucagon is limited by side effects such as hyperglycemia [206,207,208], amino acid catabolism/ureagenesis [209], and potential cardiovascular risks [210], if not carefully counterbalanced by GLP-1–mediated insulinotropic mechanisms.

Patients with glucagonoma, an alpha-cell tumor characterized by chronic glucagon hypersecretion, classically present with necrolytic migratory erythema, a blistering and migrating skin rash [211]. Notably, retatrutide is associated with dysesthesia, an abnormal and often painful skin sensation (e.g., tingling, tenderness, or sensitivity to touch or heat), affecting up to 20% of users at higher doses, a side effect not observed in tirzepatide-treated patients. This distinction raises the possibility that retatrutide-associated dysesthesia may be mediated by glucagon signaling, highlighting additional potential safety concerns related to glucagon agonism.

4. To Agonize or Antagonize the GIP Receptor?

For obesity treatment on a GLP-1 background, both GIPR agonism and antagonism can enhance weight loss; whether GIPR should be agonized or antagonized remains an active question.

Tirzepatide, a dual GIPR/GLP-1R agonist, produces ~16–22.5% weight loss at 72 weeks in people with obesity, clearly exceeding typical GLP-1RA monotherapy in SURMOUNT-1 and related trials. Long-acting GIPR agonists and GIPR/GLP-1R co-agonists reduce food intake and body weight via GIPR in inhibitory GABAergic neurons; this effect is GLP-1R-independent, so GIPR agonism adds a distinct central mechanism on top of GLP-1R pathways [212]. In mice, a GIPR/GLP-1R co-agonist (e.g., MAR709) produces greater weight loss than a PK-matched GLP-1RA, but this advantage disappears when GIPR is deleted in Vgat+ GABAergic neurons, directly linking the extra benefit to GIPR signaling in those neurons [213]. Proponents of GIPR agonism also argue that it is needed to decrease weight while mitigating the gastrointestinal adverse effects presented by GLP-1 agonists. Preclinical and early clinical data suggest that GIPR agonism may modulate GLP-1–induced nausea, and combination regimens (long-acting GIPR agonist + liraglutide) have shown improved GI tolerability compared with GLP-1RA alone [214]. Altogether, agonizing GIPR with GLP-1R leverages an extra, GLP-1R-independent central circuit (GABAergic neurons), leading to deeper weight loss and potentially better tolerability in at least some patients.

However, chronic GIPR stimulation can lead to receptor desensitization in preclinical models, and it has been hypothesized that this might functionally resemble reduced GIPR activity—an “antagonist-like” state [215]. In fact, human genetic studies show that naturally occurring loss-of-function or signaling-impaired GIPR variants are associated with lower BMI, reduced adiposity, and improved cardiometabolic profiles [216,217]. Deleting GIPR in mice also leads to less body-weight gain and fat mass accumulation on a high-fat diet, protecting them from diet-induced obesity and insulin resistance compared to wild-type controls [218]. GIPR antagonism does not appear to act through GABAergic neurons but instead modulates serotonergic circuits to enhance GLP-1R-driven anorexic effects [212]. A GIPR antagonist antibody conjugated to GLP-1 analogs (e.g., AMG-133/GIPR-Ab–GLP-1) produces greater weight loss than either GIPR antagonism or GLP-1RA alone in preclinical models [123,219]. GIPR antagonism requires intact GLP-1R signaling and acts through non-GABAergic hindbrain circuits, as it works by relieving the suppression of GLP-1 neurons [212,213]. A bispecific GIPR-Ab/GLP-1 conjugate requires both brain GIPR and brain GLP-1R for maximal weight loss, indicating that central GIPR blockade and central GLP-1R activation act cooperatively, not redundantly [220].

In summary, both GIPR agonism and antagonism can enhance weight loss, but via distinct mechanisms—GIPR agonism adds an independent GABAergic pathway, whereas GIPR antagonism boosts GLP-1R-dependent circuits. Current clinical data most strongly support dual GIPR/GLP-1R agonism (tirzepatide), while GIPR antagonism plus GLP-1R agonism is highly promising in preclinical models and early trials. The optimal choice will likely depend on individual responsiveness, side-effect profiles, and future head-to-head data rather than a single universally superior strategy.

## 12. Why Do GLP-1 Agonists Work? Weight-Loss Mechanisms and the Body-Weight Set-Point Theory

For most of the 20th century, “diet pills” were either outright toxins that harmed essential organs or near-placebos that offered little sustained benefit. The discovery of leptin (from Greek λεπτός leptos, “thin” or “light” or “small”) in the 1990s initially generated tremendous excitement as a potential cure for obesity [221]. In contrast, the identification of GLP-1 initially drew less attention. Yet, through decades of relentless biomedical engineering, most notably by Eli Lilly and Novo Nordisk, GLP-1R agonists have emerged as the most effective weight-loss medications to date. Their primary mechanism of action is appetite suppression and reduction in food intake, which allows individuals to sustain the energy deficit required for weight loss. However, the body strongly resists prolonged calorie deficits. After initial weight loss, appetite rises and resting energy expenditure falls. This phenomenon is often explained by the body-weight set-point theory, which proposes that biological processes defend a genetically determined weight range [222]. Historical work such as the Minnesota Starvation Experiment vividly demonstrated the psychological and physiological toll of chronic caloric restriction, underscoring how powerfully the body strives to restore its set-point [223].

GLP-1R agonists appear to blunt and slow some of these compensatory responses, making dietary restriction more tolerable and enabling weight losses exceeding 20% in many patients—levels rarely achieved with older pharmacological agents that seldom produced more than 10% loss. In contrast, other satiety hormones, such as PYY and cholecystokinin (CCK), only reduce meal size transiently, which is relatively easy for homeostatic systems to counter. Attempts to drive weight loss by increasing energy expenditure alone have generally fallen short: exercise provides undeniable metabolic benefits but rarely leads to substantial, sustained weight loss because increased physical activity is often offset by compensatory eating. The key determinant of long-term weight reduction is not the initial magnitude of the energy deficit but the ability to maintain it over time, a process influenced by the defended set-point [223,224].

During weight loss, most of the mass lost comes from adipose tissue. Adipose tissue itself plays a central role in body-weight regulation, as shown by animal models with genetic modifications that cause either profound obesity or lipodystrophy. Mobilization of stored triglycerides as free fatty acids is required for fat loss, and there is growing interest in whether the physical properties of adipose tissue, such as extracellular matrix rigidity or fibrosis, affect the response to pharmacologic therapy. The liver is increasingly recognized as another key regulator of systemic energy balance and may lead to adipose tissue loss through dynamic liver-adipose tissue crosstalk. Chronic glucagon signaling via hepatic cAMP pathways increases energy expenditure and results in significant weight loss in preclinical studies [225]. Incorporating glucagon receptor agonism is increasingly popular in the design of next-generation MRAs.

However, what determines this set-point is still largely unknown. Current theories suggest that it is not dictated by a single organ or molecule but instead emerges from an integrated, multi-organ network that includes the hypothalamus, adipose tissue, liver, pancreas, gut, and even skeletal muscle. Neural circuits in the arcuate nucleus, particularly proopiomelanocortin (POMC) and Agouti-related peptide (AgRP) neurons, play a central role in sensing hormonal and nutrient signals and orchestrating behavioral and metabolic responses. Yet these neurons are themselves shaped by developmental programming, genetics, inflammation, and prior weight history. Circulating signals such as leptin, insulin, FGF21, ghrelin, and GLP-1 interact with these neural pathways, but none of them alone has been sufficient to override the defended weight range without pharmacologic amplification.

Emerging evidence also points to peripheral factors that may influence the set-point, including adipose-tissue remodeling, hepatic metabolic flexibility, bile-acid signaling, microbiome composition, and even immune cell tone within metabolic organs. Chronic overnutrition appears to “reset” the defended weight upward through mechanisms such as hypothalamic inflammation, altered leptin transport, impaired nutrient sensing, and structural changes in key neural circuits. Conversely, it remains unclear whether prolonged normalization of metabolic signals—through sustained weight loss, GLP-1R agonists, combination incretin therapies, or lifestyle interventions—can durably lower the set-point or simply suppress its defenses as long as pharmacotherapy is present.

Thus, the defended body-weight set-point likely reflects a dynamic equilibrium shaped by genetics, early-life exposures, environmental inputs, and ongoing metabolic and neuroendocrine feedback. Understanding how this equilibrium is maintained and how it can be safely and durably modified remains one of the central challenges in obesity research and a critical frontier for next-generation therapeutics.

## 13. How Can We Make Them Work Better? Are There Any Other Metabolic Hormones That Can Be Combined with GLP-1 Agonists?

Appetite and energy balance are regulated by a distributed network of partially redundant and partially specialized peripheral and central pathways, rather than a single dominant axis. Interindividual variability in weight-loss response to GLP-1R agonists and current dual or triple incretin–glucagon agonists suggests heterogeneity in pathway sensitivity across patients [226]. Adding receptor agonism beyond GLP-1R, GIPR, and GCGR may enhance efficacy by engaging parallel anorexigenic or energy-expenditure pathways, compensating for reduced responsiveness of individual receptors, and enabling synergistic signaling at the level of neural circuits and peripheral organs (Table 5). Broadening the receptor repertoire is therefore a rational strategy to increase both the magnitude and consistency of therapeutic responses, rather than relying solely on higher doses of existing pathways (Table 5).

Among add-on satiety hormones, amylin has the strongest clinical support as a GLP-1 partner, offering deeper weight loss while largely recapitulating GLP-1-like gut–brain mechanisms. Amylin complements GLP-1 by slowing gastric emptying, inducing satiety, and suppressing postprandial glucagon [227]. Amylin is often paired with calcitonin because the amylin receptor is composed of the calcitonin receptor, which teams up with helper proteins (RAMPs 1–3), creating opportunities for dual amylin/calcitonin receptor activation. Beyond its role in bone, calcitonin may increase energy expenditure and activate satiety neurons [228,229]. CagriSema, a co-formulation of an amylin analog with semaglutide, produced weight loss in the low-20% range and efficacy comparable to high-dose semaglutide but did not meet non-inferiority versus tirzepatide 15 mg in the head-to-head trial (NCT06131437), highlighting both the promise and the competitive bar for amylin-based combinations.

Additional satiety-axis strategies seek to stack multiple gut–brain signals on top of GLP-1. CCK from intestinal I-cells slows gastric emptying, promotes satiation, and supports insulin secretion [230], prompting the development of dual GLP-1/CCK analogs. PYY from L-cells reduces appetite via hypothalamic Y2 receptors [231,232,233] and acts additively with GLP-1 on gastric emptying and food intake, and lower-dose PYY analogs are being co-administered with GLP-1RAs (for example, Nisotirostide with semaglutide, tirzepatide, or dulaglutide) to improve tolerability. Leptin, secreted in proportion to fat mass [234], is a key satiety and energy-expenditure hormone [235], but leptin resistance has limited its standalone use [236], prompting dual GLP-1/leptin and GLP-1/glucagon approaches plus antibody-based leptin agonists to restore responsiveness. In contrast, antagonizing the orexigenic hormone ghrelin, via GHSR blockade or LEAP2-like antagonists, may curb hedonic eating and improve insulin sensitivity [237,238], though concerns such as hypoglycemia, depressive-like behavior, and sex-dependent responses have slowed translation [239,240,241,242]. Together, CCK, PYY, leptin, and ghrelin-axis strategies exemplify complementary satiety pathways that may amplify GLP-1–driven weight loss and, if carefully tuned for tolerability, could contribute to more durable changes in the defended body-weight set-point.

Beyond satiety, hormones and pathways that reshape nutrient partitioning and organ crosstalk, such as FGF21, PPARs, IGF-1, and related muscle–bone axes, are especially promising for improving the quality of weight loss, with early data suggesting disproportionate benefits on liver fat, dyslipidemia, and lean mass preservation. Multi-receptor designs that extend beyond GLP-1/GIP/GCG toward liver, adipose, and skeletal muscle targets may ultimately be required to meaningfully shift the defended body-weight set-point, rather than merely enabling a deeper but still transient calorie deficit. Most other hormonal combinations, including MC4R, apelin, and many next-generation constructs, remain early-stage and are best viewed as probes that map the therapeutic “design space” rather than validated add-ons ready to rival current incretin-based MRAs; their modest efficacy as monotherapies likely reflects non-optimal molecule design and the limits of single-axis therapy and does not exclude additive or synergistic benefits when layered onto GLP-1RAs.

**Table 5:** Physiological levels and clinical development of endogenous hormones combined with GLP-1R agonists.

Hormone	Secretion Source	Fasting Concentration (Human)	Fed/Post-Meal Concentration (Human)	Primary Physiological Effects	Current Regulatory and Clinical Status
GIP	Duodenal K-cells [243]	~3–18 pM (fasted) [244]	~22–160 pM (post-meal) [244]	↑ Insulin (glucose-dependent) ↑ Fat storage [245]	Standalone: Experimental With GLP-1: Approved for Obesity/T2D (Tirzepatide) [13]
Glucagon	Pancreatic α-cells [246]	~10–55 pM (fasted) [244]	~10–65 pM (post-meal) [244]	↑ Blood glucose (glycogenolysis/gluconeogenesis) ↑ Lipolysis [244]	Standalone: Approved for Hypoglycemia Rescue With GLP-1: Phase 2/3 [35,247]
Amylin	Pancreatic β-cells [248]	~2–10 pM (fasted) [249]	~3–11 pM (post-meal) [249]	↑ Satiety ↓ Gastric emptying ↓ Postprandial glucagon [46]	Standalone: Approved for T1D/T2D (Pramlintide) With GLP-1: Phase 2/3 [117,120,250]
OXM	Intestinal L-cells [248]	~10–30 pM (fasted) [251]	~12–20 pM (post-meal) [251]	↑ Satiety ↑ Energy expenditure [252]	Standalone: Approved for Obesity/T2D in China (Mazdutide)
PYY	Intestinal L-cells [248]	~7–40 pM (fasted) [244]	~7–30 pM (post-meal) [244]	↓ Appetite (“ileal brake”) ↓ Gastric emptying [253]	Standalone: Experimental With GLP-1: Phase 1/2 [254]
Ghrelin	Stomach (mainly) [248]	~100–200 pM (fasted) [255]	↓ to ~50 pM (post-meal) [256]	↑ Hunger ↑ Growth hormone release ↑ Gastric motility [248]	Standalone: Experimental With GLP-1: N/A [257]
CCK	Duodenal/Ileal I-cells [243]	~1–5 pM (fasted) [258]	~5–30 pM (post-meal) [258]	↑ Bile/enzyme release ↑ Satiety ↓ Gastric emptying [253]	Standalone: Approved as Diagnostic Agent (Sincalide) With GLP-1: Experimental [259]
Leptin	Adipose tissue	~1–10 ng/mL (fasted) [256,260]	↓ with fasting [256,260]	↑ Long-term satiety signal ↓ Food intake (via hypothalamus) [261]	Standalone: Approved for Lipodystrophy (Metreleptin) With GLP-1: Experimental [262,263]
FGF21	Liver, adipose [248]	~1–26 pM (fasted) [264]	↑ with fasting/ketosis [265]	↑ Insulin sensitivity ↑ Fatty acid oxidation ↑ Ketogenesis [266]	Standalone: Phase 2/3 With GLP-1: Phase 2 [267]
Calcitonin	Parafollicular C-cells of thyroid gland [268]	~1.9 pM [269]	↑ with fasting (post-meal) [269]	↓ Blood calcium levels ↓ Bone resorption [270]	Standalone: N/A With GLP-1: Experimental [271]
Apelin	Endothelial cells, adipose tissue, GI tract, CNS [272]	~45–85 pM (fasted) [273]	↑ with fasting (post-meal) [274]	↑ Glucose uptake, improves insulin sensitivity, vasodilation, cardiovascular effects	Standalone: Experimental With GLP-1: Experimental [275]

**↑** indicates an increase of the parameter following the symbol. **↓** indicates a decrease.

## 14. One of the Many Challenges to Overcome: To Predict Efficacy Early

One of the major challenges in the development of next-generation GLP-1R agonists, or in drug discovery in general, is predicting efficacy early in the drug discovery process [276].

While traditional GLP-1R agonists have established clinical success in glycemic control and weight reduction [277], the ability to forecast efficacy across new molecular scaffolds remains limited [278]. The pharmacological effectiveness of a GLP-1 agonist is largely determined by its potency (e.g., EC_50_), pharmacokinetic half-life, and receptor engagement format [279]; however, these parameters alone often fail to predict long-term outcomes observed in humans [280,281,282].

The challenge becomes even greater with dual and triagonists that target multiple incretin pathways [276,283]. In such cases, interspecies differences can complicate preclinical interpretation—for instance, the GIPR is poorly conserved between mice and humans, making receptor humanization a critical step in early efficacy determination [284]. Without such precautions, promising candidates may be mischaracterized during preclinical screening.

Moving forward, integrating pharmacology, molecular profiling, and receptor modeling data may improve predictive power during early development. Yet, even with optimized preclinical systems, translation to clinical efficacy remains uncertain [276]. Biological and socioeconomic variables can affect treatment adherence and persistence rates, which may hinder long-term cost-effectiveness and widespread use of these medications even if effective [285,286]. In human studies, weight-loss outcomes are also strongly confounded by the study population’s diet, eating patterns, activity levels, and lifestyle adherence, all of which introduce substantial variability and make predictive modeling increasingly difficult [287,288,289,290].

Integrating artificial intelligence, precision medicine, and pharmacogenomics can help synthesize these complex variables to forecast individual treatment responses. With increased predictive modeling for incretin sensitivity and tolerability, high interindividual variability can subside in future clinical trials [291,292].

## 15. Conclusions

Current evidence supports that GLP-1-based therapies can suppress defended weight mechanisms, enabling substantial weight loss before a new plateau is reached. However, the quality of this weight loss, particularly the balance between fat loss and preservation of lean mass, will determine long-term metabolic and functional outcomes. Emerging multi-receptor agonists and combination approaches that add additional metabolic hormone components offer a promising path to increase weight loss while improving body-composition profiles. Yet, fundamental questions remain about how these interventions reshape the defended body-weight set-point over years, underscoring the need for long-term, mechanistically informed studies that integrate adiposity, organ-specific effects, and muscle preservation.

## Data Availability

No new data were created or analyzed in this study. Data sharing is not applicable to this article.
